# Perception and action as viewed from the Theory of Event Coding: a multi-lab replication and effect size estimation of common experimental designs

**DOI:** 10.1007/s00426-022-01705-8

**Published:** 2022-08-17

**Authors:** Markus Janczyk, Carina G. Giesen, Birte Moeller, David Dignath, Roland Pfister

**Affiliations:** 1 Department of Psychology, University of Bremen, Bremen, Germany; 2 Institute of Psychology, Friedrich Schiller University Jena, Jena, Germany; 3 Cognitive Psychology, University of Trier, Trier, Germany; 4 Department of Psychology, Eberhard Karls University of Tübingen, Tübingen, Germany; 5 Department of Psychology III, University of Wuerzburg, Würzburg, Germany

## Abstract

The Theory of Event Coding (TEC) has influenced research on action and perception across the past two decades. It integrates several seminal empirical phenomena and it has continued to stimulate novel experimental approaches on the representational foundations of action control and perceptual experience. Yet, many of the most notable results surrounding TEC originate from an era of psychological research that relied on rather small sample sizes as judged by today’s standards. This state hampers future research aiming to build on previous phenomena. We, therefore, provide a multi-lab re-assessment of the following six classical observations: response-effect compatibility, action-induced blindness, response-effect learning, stimulus–response binding, code occupation, and short-term response-effect binding. Our major goal is to provide precise estimates of corresponding effect sizes to facilitate future scientific endeavors. These effect sizes turned out to be considerably smaller than in the original reports, thus allowing for informed decisions on how to address each phenomenon in future work. Of note, the most relevant results of the original observations were consistently obtained in the present experiments as well.

## Introduction

Good experiments test predictions derived from theory. Albeit integral to scientific psychology, recent discussions have questioned whether experiments actually hold up to this standard (Shrout & Rodgers, 2018). One reason for this is the allegedly poor reproducibility of some experimental results (see, most prominently, Open Science Collaboration, 2015), and, consequently, several projects aimed to increase the reproducibility and robustness of empirical phenomena (Many Labs: Klein et al., 2014; the Psychological Science Accelerator: Moshontz et al., 2018; Registered Replication Reports: Simons et al., 2014). Two features characterize these approaches. First, they typically focus on a binary distinction between “replicating” versus “not replicating” phenomena that had been reported in earlier work. Second, if targeting more than a single phenomenon of interest, the selection of studies for replication in these approaches has mostly been guided by pragmatic and methodological reasons (i.e., to avoid selection bias). Yet, more and more researchers argue that not (only) limited reproducibility as such, but rather limited theorizing is an important factor for the current ‘crisis’ in psychology, that is, a lack of cumulative theory building and integrative frameworks going beyond phenomena-specific explanations (Eronen & Bringmann, 2021; Muthukrishna & Henrich, 2019; Oberauer & Lewandowsky, 2019).

The present research contributes to the aim of cumulative theory-building not by asking whether or not certain phenomena of interest exist, but rather by providing a conjoint re-assessment and specification of a set of related empirical phenomena pertaining to one and the same theoretical framework. By increasing precision of corresponding effect size estimates for several related and influential observations, these results will allow for continued investigation and thus cumulative and informed theorizing.

The topic of interest of the present study was the basic interplay of perception and action as captured by the Theory of Event Coding (TEC; Hommel et al., 2001)—an influential theoretical framework linking a wide range of research on the representational foundations of perceptual coding and action control. To determine a maximally informative set of empirical phenomena, we invited multiple experts (determined on the basis of previous publications) in the field of cognitive psychology to indicate the most important phenomena for TEC and TEC-inspired research. Based on this expert consensus, we conducted high-powered, multi-lab replications of six particular phenomena with the goal of obtaining precise estimates of the corresponding effect sizes. Considering multiple phenomena appears important to us, as theories can be construed as generalizations aiming to link different phenomena (Borsboom et al., 2021; Gozli, 2019). Going beyond the binary distinction of replication versus failure-to-replicate, providing precise effect-size estimates further provides a common ground for future experimentation in the field, because such effect size estimates are pivotal for informed power calculations (Brysbaert, 2019).

In the following section, we provide a short historical background, followed by an overview of the basic tenets of TEC. Thereafter, we introduce the motivation of the present study in more detail.^1^

### Theoretical approaches to human perception and action

Perception and action are the cornerstones of psychological inquiry, and perceptual processing as well as action-related decision making have enjoyed widespread interest throughout most of psychology’s history. In contrast, the mechanics of how decisions and action intentions are actually translated into overt motor behavior has traditionally received only little attention in psychological research, and this research area has, therefore, been dubbed as the “Cinderella of Psychology” (Rosenbaum, 2005). In fact, motor processes have been often viewed as the mere endpoint of a processing stream, echoing basic ideas of early philosophical accounts of perception and action (Descartes, 1664; Dewey, 1896; see also Hommel & Elsner, 2000).^2^ This view of motor processes as the final, operative end of psychological processing is particularly explicit in stage theories of human information processing (Donders, 1868; see also Pashler, 1994; Sanders, 1990). These theories assume a processing stream beginning with perception of a stimulus. In a subsequent stage, often referred to as response selection, the resulting perceptual representation is translated into a motor representation which is used to emit the respective motor behavior, rendering the motor part a vicarious, subordinate process. The question of how cognitive processes actually interface with the agent’s motor repertoire, however, is commonly not considered by these models.

The theoretical stance adopted by information processing accounts mainly derived from a common focus on inferring processing characteristics—such as potential capacity limitations—rather than tackling how action plans are represented in the human cognitive system (but see Janczyk & Kunde, 2020, for a specification related to TEC). This state of affairs changed quite drastically with the rise of research that was stimulated by the hypothesis of common coding of perception and action (Prinz, 1984, 1992; see also Prinz et al., 2013).

Research that implemented the common coding framework was driven by the ambitious goal of determining the representational foundations of human action control (Prinz, 1984). Common coding breaks with traditional views that see perception and action as two distinct compartments of cognitive function—an assumption that psychological research had inherited from its roots in Western philosophy and its Cartesian view of the mind (Descartes, 1664). Neurophysiological and neuroanatomical approaches had followed the same route by portioning the brain in “sensory” and “motor” compartments (e.g., Harleß, 1861; Laycock, 1845; see also Pfister & Janczyk, 2012). Accepting a qualitative divide between perception and action requires additional theorizing to explain how actions are brought about, for example, by introducing concepts such as stimulus–response translation or response selection as mentioned above. As an elegant and powerful alternative, common coding suggests that perception and action planning share the property to internally represent events in a commensurable representational format, that is, in a common code. The notion of common coding therefore explains a range of direct effects of perception on action planning and vice versa, and it allows for modelling action planning and initiation as anticipated perception (Hommel, 2009), a notion borrowed from the ideomotor principle of human action control (Harleß, 1861; James, 1890). These ideas were summarized in a landmark article that introduced TEC (Hommel et al., 2001). TEC has had a considerable impact on psychological research in this field ever since, and we describe its major tenets in the following section.

### The Theory of Event Coding (TEC)

Perception and action control involve many layers of physiological and cognitive processes. Perception for instance, will always involve basic receptors, such as the photoreceptors in the retina that transmit signals for further processing. Crucially, these sensory signals encode information about entities in the world (objects, people, the agent’s own body), and representations of these entities are constructed by processing certain aspects of the sensory data (Brunswik, 1944; Heider, 1930/1959; Prinz, 1992). TEC is concerned with these latter representations, that is, representations in terms of distal codes. Proximal events, such as activity of sensory receptors or muscle physiology, are only relevant in that they interface with distal codes and form the basis of such distal representations (Hommel, 2009; Hommel et al., 2001).^3^ It is because actions rely on anticipations of to-be produced perceptions that the level of distal representations prepares the ground for a common representational format in which perception and action control can interact (Prinz, 1992).

TEC further follows previous work on (visual) perception and motor planning in assuming that perceptual events and action plans alike are composed of individual features that have to be bound together to form integrated representations (Allport, 1993; Rosenbaum, 1980; Treisman, 1996). In TEC’s terminology, such feature bundles are referred to as *event files* (Hommel, 2004; Hommel & Wiers, 2017). If a feature becomes activated, it spreads activation to the event that it is associated with. Once an event is fully retrieved or established by integrating the corresponding features into an event file, this representation will inhibit competing event files that would draw on features that are now less available. This dynamic interplay of activation and integration is a core mechanism to explain why several perceptual representations and action representations sometimes boost each other while they interfere at other times (see also Thomaschke et al., 2012).

But why would an organism form such integrated event files at all? A major consequence of the proposed cognitive architecture is that activating features allows for selecting, planning, and initiating overt bodily movements. Here, TEC imports ideomotor theorizing in assuming that actions are coded in terms of the sensory consequences they produce (Hommel, 2009). These sensory consequences can relate to perceived movements of the agent’s body and perceived changes in the agent’s environment alike, as long as they are consistently related to certain motor actions (Pfister, 2019). Crucially, TEC assumes that activating sufficiently many features of such an action is the cognitive antecedent of overt bodily movements in that these representations are bi-directionally related to activity of the muscular system (Hommel, 2009; Hommel & Wiers, 2017). This assumption thus closes the gap between perception and action and allows TEC to capture the cognitive underpinnings of perceptual processing and action planning alike. It, therefore, offers a theoretical connection between several areas of psychological theorizing, ranging from multisensory integration to learning and motor control. Each of these fields, of course, comes with its own theories tailored to understanding relevant key findings (e.g., Ernst & Bülthoff, 2004; Jeannerod, 2006). What makes TEC unique, however, is that it provides a framework for distilling commonalities between these seemingly distinct aspects of human cognition. Beyond this basic research interests, aspects of TEC have further been used as a framework in applied research as well, for example, in clinical research (Kleimaker et al., 2019; Petruo et al., 2019).

### Goals of the present study

TEC draws on a large array of experimental approaches that address interesting questions on the interplay of perception and action. As such, it has stimulated a remarkable range of novel discoveries. Most of the original studies, however, originate from a period of psychological research in which power calculations had not arrived in the methodological mainstream and small sample sizes were thus the norm (*n* = 8–18 in the main studies considered below). Certainly, this does not compare favorably with current requirements and standards (Brysbaert, 2019), and this empirical limitation has several direct consequences for current research.

As a first consequence, small sample sizes provide little power for detecting effects of interest if corresponding effect sizes are small, and they provide little grounds for judging the absence of an effect as compared to the mere absence of evidence in favor of this effect. Several relevant theoretical claims do rest on such null effects, however. For example, Kunde (2001, Exp. 1) took a non-significant effect to infer that different mappings of responses to response-contingent effects can be acquired flexibly without being overshadowed by previous associations including the same events. Another example relates to the study by Hommel (1998), who bases a key argument on the absence of higher-order interactions in the corresponding experimental design. To evaluate the empirical foundations of such claims, it is instructive to ask whether such studies would have had sufficient power to detect small effect sizes that would be theoretically relevant nevertheless. Here, the study by Kunde (2001) came with a power of about 1 − *β* = .05 to detect an effect size of Cohen’s *d*_s_ = 0.2 in the corresponding between-participants comparison (*n* = 10 overall, distributed equally across both sequence conditions), and the study by Hommel (1998) came with a similar power of 1 − *β* = .07 to detect relevant within-participant effects of Cohen’s *d*_*z*_ = 0.2 (*n* = 8). Conversely, the former study would have to assume Cohen’s *d*_s_ = 2.0 for a satisfactory power of 1 − *β* = .80 and the latter study would have to assume Cohen’s *d*_*z*_ = 1.2 for the same power, so that sufficient power is only available for considerably large effects. Whether or not the corresponding theoretical claims hold is thus difficult to assess based on these original studies.

As a second consequence, small sample sizes tend to yield effect size estimates that are heavily biased and often inflated (see Ulrich et al., 2018). In this context, it should be noted that the presence versus absence of an effect is critical for TEC as described in the preceding paragraph, whereas the exact size of an empirical effect does not have theoretical implications per se. As TEC continues to stimulate novel research, however, it would be highly useful for the community to have reliable estimates of how big certain effects are when planning sample sizes via power calculations. If such power calculations rely on inflated estimates of the population effect sizes, the resulting studies will be underpowered and thus are easily subject to not detecting an effect that is actually present. Such null effects might either end up as unpublished file-drawer results; worse, they might be misunderstood as another example of non-reproducible phenomena or they might even motivate incorrect theorizing when taken to indicate absence of an effect. In this regard, it seems notable that there are no direct replications of the original experiments in the literature, despite a striking number of conceptual replications and extensions. Within these conceptual replications, effect size estimates also vary considerably at times (e.g., ranging from *d*_*z*_ = 0.37 to *d*_*z*_ = 0.99 in a re-analysis of the data reported by Pfister et al., 2010), suggesting that some of the findings might relate to underpowered designs. Considering the precision of effect sizes is thus both of practical and theoretical relevance. For instance, moderation and mediation hypotheses require knowledge about the heterogeneity of effect sizes, because they ask questions about relative changes in the size of effects. Similarly, fields such as personality and clinical psychology ask questions about the interindividual variation of effect sizes.

The present study aims to solve these limitations by re-assessing the empirical basis of TEC with experiments using sufficiently large samples. We will describe the corresponding methodological considerations in the following section.

## Study selection

TEC is grounded in a diverse range of empirical phenomena and, in turn, it has stimulated a remarkable range of experimental approaches. Necessarily then, any approach to re-assessing the empirical foundation of TEC-related effects has to be selective. In this case, study selection proceeded in the following two steps: a first step to determine the most relevant empirical phenomena, and a second step to determine the exact experimental design to assess the phenomena of interest.

### Empirical phenomena

A challenge for determining the most relevant empirical phenomena for TEC is that the theoretical relevance of different phenomena certainly comes with a subjective component so that it may be gauged differently across researchers. Moreover, several phenomena have been picked up by distinct communities, depending on their preferred theoretical focus. We therefore chose to base our selection not on our own preferences, but rather opted to involve a large number of experts in the field to strive for a maximally representative selection. We reached out to 114 researchers who had published studies with related observations and asked them to rate which empirical phenomena and corresponding approaches they perceived as closely tied to TEC, either by lending considerable support to its theoretical notions, or by being immediately stimulated by TEC. More precisely, we asked for a structured response in a brief questionnaire (see Appendix A) and received a total of 49 responses (including our own assessment). The questionnaire was online for one month after its dissemination via e-mail in September, 2018.

This questionnaire included the following ten items in alphabetical order: action-induced blindness (e.g., Müsseler & Hommel, 1997a), code occupation (e.g., Stoet & Hommel, 1999), dimension weighting (e.g., Fagioli et al., 2007), distractor-response binding (e.g., Frings et al., 2007), feature weighting (e.g., Memelink & Hommel, 2013), response-effect (R-E) compatibility (e.g., Kunde, 2001), R-E learning (e.g., Elsner & Hommel, 2001), sensory attenuation (e.g., Blakemore et al., 2000), short-term R-E binding (e.g., Dutzi & Hommel, 2009), and stimulus–response (S-R) binding (e.g., Hommel, 1998).

The questionnaire further provided the opportunity to comment on our selection and to suggest additional phenomena that were not included in the original list. In addition to elaborate theoretical remarks, several such phenomena were brought up by the experts, including the impact of action effects on S-R compatibility phenomena (Hommel, 1993), referential coding (Dolk et al., 2013), longer-term associations between stimuli and responses such as instances (Logan, 1988), levels of S-R representations (Horner & Henson, 2011), and stimulus-task bindings (Waszak et al., 2003). Their clear relation to TEC notwithstanding, none of these phenomena was brought up by more than one expert so that we chose to retain our original selection of ten phenomena.

**Fig. 1:**
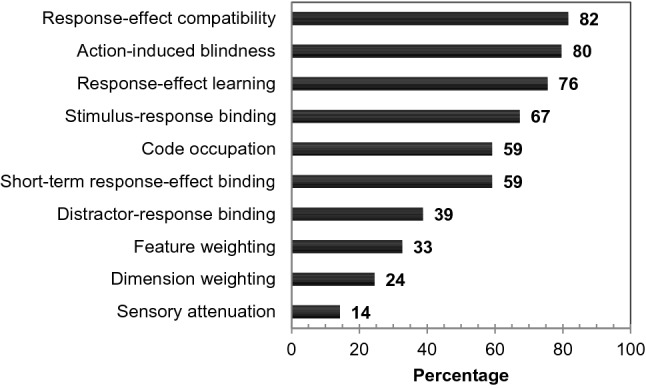
Percentages of affirmative responses to the question on which empirical phenomena and approaches are related particularly closely to TEC, either by lending considerable support to its theoretical notions, or by being immediately stimulated by TEC. Percentages are relative to the total number of the 49 expert responses

For each of the ten empirical phenomena, we assessed how many of the respondents had marked them as highly related to TEC. Figure 1 summarizes the resulting opinions. Six of the ten phenomena were suggested by more than 50% of the experts, so that we settled on these phenomena as a final selection: R-E compatibility (82%), action-induced blindness (80%), R-E learning (76%), S-R binding (67%), code occupation (59%), and short-term R-E binding (59%).

### Experimental designs

The selection of the exact experimental design we used in the present study was based on three criteria. First, we considered always the first study on the particular effect of interest as judged by the date of the corresponding journal publication. Second, we opted for taking the first reported experiment in a study that used a within-subject design for the effect of interest. Third, we took into account the possibility to run the experiment on standard computer hardware without specific display or input devices, to be able to run the same experiment in different labs. From these considerations, Experiment 1 was chosen for the studies by Dutzi and Hommel (2009), Kunde (2001), Müsseler and Hommel (1997a), and Stoet and Hommel (1999). For the study by Elsner and Hommel (2001), we chose Experiment 3a for the following two reasons: Experiment 1 would have involved a between-subject comparison, and Experiment 2—although using a within-subject comparison—did not employ catch trials in a free-choice task, a point we considered a shortcoming of this experiment. Finally, from the study by Hommel (1998), we focused on Experiment 1b, thus omitting the single-response condition used in Experiment 1a.

## Empirical approach and main results

### Sample sizes and participants

The goal of our study was to improve the precision of effect size estimates for a range of experimental effects. We, therefore, could not rely on traditional power calculations, because the resulting sample sizes are optimized for deciding whether or not a non-zero effect is present in the population, given an effect of particular size. Instead, we planned our sample size according to how precisely effects can be estimated, operationalized as the width of the 95% confidence interval for standardized effect sizes.

A detailed account of this approach is provided in Appendix B. This approach suggests a sample size of *n* = 100 participants to yield such confidence intervals with a width of 0.4, thus allowing to characterize the resulting effect size at a precision of roughly ± 0.2 (note that confidence intervals for standardized means are not perfectly symmetrical).

With this in mind, we decided to recruit a total sample of *n* = 120 participants to be able to compensate potential data loss. As per our pre-registration (https://aspredicted.org/ad6xq.pdf), data from *n* = 30 participants were collected in each of four different laboratories in Freiburg, Jena, Trier, and Würzburg (all Germany).^4^ Table 1 summarizes the demographics for the entire sample and the subsamples. An Analysis of Variance (ANOVA) on the participants’ age with the between-participants factor laboratory site was significant, *F*(3, 116) = 14.33, *p* < .001, $\eta_{{\text{p}}}^{2}$ = .27. The gender distribution did not differ between laboratories, *χ*^2^(6) = 8.30, *p* = .217, and the same was true for handedness, *χ*^2^(3) = 3.02, *p* = .389.

**Table 1:** Summary of demographic variables for the total sample and separately for the subsamples for which the data was collected in different laboratories

Lab	*n*	Age		Gender			Handedness	
		Mean (SD)	Range	Female	Male	Non-binary	Right	Left
Freiburg	30	23.8 (3.7)	19–35	22	8	0	29	1
Jena	30	21.3 (4.1)	18–38	24	5	1	27	3
Trier	30	21.6 (2.0)	19–28	28	2	0	25	5
Würzburg	30	28.8 (8.1)	20–52	22	8	0	26	4
Total	120	23.9 (5.8)	18–52	96	23	1	107	13

If participants were excluded from analyses (due to specific criteria or because of incomplete data), this will be mentioned in the respective methods parts of the particular experiment.

### General approach

Based on the expected duration of the experiments (judged by pretests), the six experiments were grouped into two clusters. Cluster 1 comprised short-term R-E binding (Dutzi & Hommel, 2009, Exp. 1), action-induced blindness (Müsseler & Hommel, 1997a, 1997b, Exp. 1), and code occupation (Stoet & Hommel, 1999, Exp. 1); Cluster 2 comprised R-E compatibility (Kunde, 2001, Exp. 1), R-E learning (Elsner & Hommel, 2001, Exp. 3a), and S-R binding (Hommel, 1998, Exp. 1b). All participants took part in all six experiments and the two clusters were applied in two 2-h sessions, scheduled on two different days within one week. The order of experiments within a session was counterbalanced, while the order of clusters was determined randomly for each participant.

**Fig. 2:**
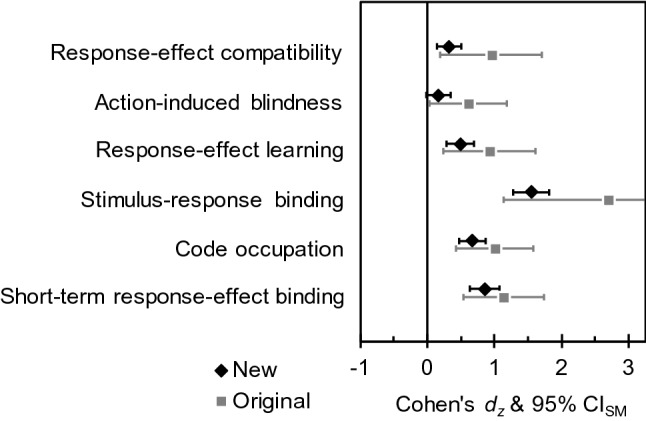
Overview of the effect sizes in the original studies and in the present experiments

Analyses and presentation of results were kept closely to the original studies. In several cases, we supplement these results with additional analyses, for example, including additional factors that were not analyzed originally or applying different criteria for participant exclusion. Crucially, we also extracted effect size estimates for each main comparison of interest, and we computed the corresponding 95% confidence interval for standardized means around this effect size estimate. Figure 2 provides an overview of the main results relative to the estimates of the original studies. Details on how we computed the original effect sizes are reported in Appendix C. The six selected studies will be described in the following, ordered according to the degree of agreement among the experts. Data and analyses scripts can be found at https://osf.io/hgy5q/.

## Experiment 1: response-effect compatibility

TEC builds on the ideomotor principle and views action planning as anticipated perception (Harleß, 1861; Herbart, 1825; James, 1890; for reviews, see Greenwald, 1970; Shin et al., 2010; for historical comments, see Pfister & Janczyk, 2012; Stock & Stock, 2004). According to the ideomotor principle, representations of an action’s consequences, often termed *action effects* in psychology, are functionally relevant not only for deciding between different behavioral options, but also for actually initiating and controlling a bodily movement (Kunde et al., 2004; Wirth et al., 2016).

Compelling evidence for the functional role of action-effect anticipation comes from experiments on R-E compatibility (Kunde, 2001). Here, arbitrary stimuli (often: visual or auditory events) are presented as effects contingent on the responses of the participants. Crucially, responses and effects vary on a shared dimension such as spatial location, temporal parameters, or intensity. For S-R compatibility experiments, such dimensional overlap (or set-level compatibility; Kornblum et al., 1990) has repeatedly been observed to yield robust compatibility effects, that is, stimuli facilitate those responses that come with compatible rather than incompatible features on the element-level of this dimension (Fitts & Deininger, 1954; Fitts & Seeger, 1953; see Tlauka & McKenna, 1998 for an extension to imagined stimuli). If action control indeed invokes sensory anticipations of upcoming action effects, as suggested by the ideomotor principle, then similar compatibility phenomena should also arise between responses and following, response-contingent effects.

**Fig. 3:**
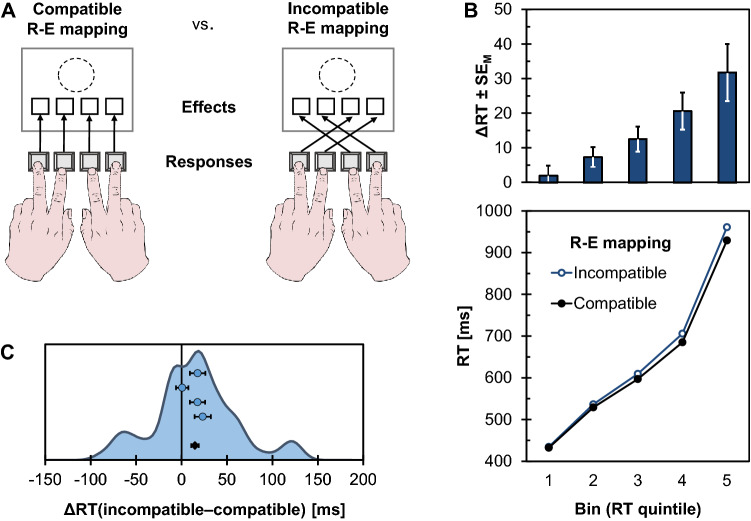
Design and results of the response-effect (R-E) compatibility experiment (Exp. 1). **A** Participants responded with horizontally aligned response keys to a colored target circle. Each keypress response predictably triggered a visual effect on the computer screen, either at a corresponding spatial location (compatible condition) or at a non-corresponding spatial location (incompatible condition). R-E compatibility conditions were manipulated between experimental halves. **B** Response times (RTs) in milliseconds (ms) as a function of RT quintile and R-E compatibility mapping, accompanied by the resulting R-E compatibility effects (ΔRT) and corresponding standard errors of the mean (SE_M_). **C** Distribution of individual R-E compatibility effects (ΔRT; shown as kernel density estimate) in RTs together with means and standard errors of the four samples (blue dots) and the pooled data (black diamond)

Figure 3A shows the setup of the initial demonstration of an R-E compatibility effect (Kunde, 2001, Exp. 1). Participants (*n* = 10) responded to the color of a target stimulus by pressing one of four horizontally aligned keys. Each key consistently triggered a visual action effect at a compatible location (e.g., a left keypress lighting up a left visual effect) or at an incompatible location (e.g., a left keypress lighting up a right visual effect). R-E mapping was manipulated between experimental halves so that participants could predict precisely which action effect would follow from their responses. Response times (RTs) in this setting were shorter in the compatible condition than in the incompatible condition, despite action effects only appearing *after* RT had been measured. Thus, because the experimental manipulation affects an event occurring after responding, these results provide compelling evidence for the idea that effect anticipations are functionally relevant for action control.

### Method

#### Responses and stimuli

Responses were given with the left and right index and middle fingers on the keys ‘d’, ‘v’, ‘n’, and ‘k’. Centrally presented red, green, blue, and yellow circles were used as stimuli (presented against a black background) and were mapped to the response keys in this order from left to right for all participants. Outlines of four rectangles were presented below screen center throughout the whole experiment and served as placeholders for the visual action effects. One of the squares was filled white as the action effect.

#### Task and procedure

The participants’ task was to press the correct response key according to the stimulus color. A trial began with an auditory warning click (1000 Hz, 20 ms; the warning click was 100 Hz in the original study, but we preferred to use a higher frequency to achieve a clearer sound independent of the hardware). The stimulus appeared after 500 ms and remained on screen until a response was given (a trial was aborted when a response was given prior to stimulus onset). The action effect appeared immediately following the response and remained on screen for 300 ms. In case of errors, written feedback was presented for 500 ms above the screen center. The next trial started after an inter-trial interval (ITI) of 1000 ms. In compatible blocks, the action effect occurred in the square spatially corresponding to the response; in incompatible blocks, the action effect occurred in a square that was shifted two positions to the left (for right hand responses) or to the right (for left hand responses).

The whole experiment comprised 30 blocks with 16 trials each. For half of the participants, the first 15 blocks were with a compatible R-E mapping, and the subsequent blocks with an incompatible R-E mapping. This order was reversed for the other half of participants. Instructions emphasized both speed and accuracy and mentioned that a compatible or incompatible square would be filled following each response.

#### Design and analyses

All participants were included into the analyses. Trials with responses prior to stimulus onset were excluded first. RTs were measured from stimulus onset until the response. Only correct trials were used for RT analyses and trials with RTs < 200 ms or > 1500 ms were excluded as outliers (2.49%).

For the first analyses, the following two independent variables were of interest: (1) R-E mapping as a repeated-measure (R-E mapping: compatible vs. incompatible) and (2) order as a between-participants variable (order: compatible-incompatible vs. incompatible-compatible). Order was included for comparability with the original analyses. Mean correct RTs and percentages of errors (PEs) were analyzed with the according mixed ANOVA. For the second analysis, correct RTs were split into five quintiles, separately for each R-E mapping condition and participant. Data were then submitted to a 2 × 5 ANOVA with R-E mapping and bin as repeated-measures.^5^ In case the sphericity assumption was violated, the Greenhouse–Geisser correction was applied and the corresponding ε is reported. Separate paired *t*-tests were computed for each bin to assess the R-E compatibility effect separately. Individual R-E compatibility effects were calculated as ΔRT = RT_incompatible_ – RT_compatible_.

### Results

Mean correct RTs and PEs are summarized in Table 2, and Fig. 3 shows detailed descriptive statistics for the quintile analysis (Fig. 3B) and the distribution of individual R-E compatibility effects (Fig. 3C). For RTs, the main effect of R-E mapping was significant, *F*(1, 118) = 13.03, *p* < .001, $\eta_{{\text{p}}}^{2}$ = .10. In addition, participants responded faster with the order ‘incompatible-compatible’ than with the opposite order, *F*(1, 118) = 5.28, *p* = .023, η_p_^2^ = .04. The interaction was not significant, *F*(1, 118) = 2.67, *p* = .105, $\eta_{{\text{p}}}^{2}$ = .02. Averaged across both orders, the R-E compatibility effect was 15 ms (see Fig. 3C), *t*(119) = 3.58, *p* < .001, *d* = 0.33, 95% CI_SM_ = [0.14, 0.51].

**Table 2:** Mean correct response times (RTs) in milliseconds (ms) and percentages of errors (PE) as a function of order and response-effect (R-E) relation for Experiment 1

	RTs [ms]		PE	
	R-E Relation		R-E Relation	
Order	Compatible	Incompatible	Compatible	Incompatible
Compatible—incompatible	650	671	6.11	6.13
Incompatible—compatible	620	628	4.86	4.63

Participants made overall more errors with the order ‘compatible-incompatible’, although the main effect was not significant, *F*(1, 118) = 3.69, *p* = .057, $\eta_{{\text{p}}}^{2}$ = .03. The main effect of R-E mapping was not significant, *F*(1, 118) = 0.25, *p* = .619, $\eta_{{\text{p}}}^{2}$ < .01, and the same was true for the interaction, *F*(1, 118) = 0.32, *p* = .570, $\eta_{{\text{p}}}^{2}$ < .01.

Mean correct RTs are visualized in Fig. 3B separately for the five RT quintiles. The corresponding ANOVA revealed a significant main effect of R-E mapping, *F*(1, 119) = 13.03, *p* < .001, $\eta_{{\text{p}}}^{2}$ = .10, and of bin, *F*(4, 476) = 1740.33, *p* < .001, $\eta_{{\text{p}}}^{2}$= .94, ε = .295. In addition, the interaction was significant, indicating an increase of the R-E compatibility effect with increasing RTs, *F*(4, 476) = 13.07, *p* < .001, $\eta_{{\text{p}}}^{2}$ = .10, *ε* = .361. The R-E compatibility effect was significant from the second quintile on, Quintile 1: *t*(119) = 0.71, *p* = .478, *d* = 0.06; Quintile 2: *t*(119) = 2.61, *p* = .010, *d* = 0.24; Quintile 3: *t*(119) = 3.52, *p* = .001, *d* = 0.32; Quintile 4: *t*(119) = 3.85, *p* < .001, *d* = 0.35; Quintile 5: *t*(119) = 3.84, *p* < .001, *d* = 0.35.

### Discussion

As in the original demonstration of an R-E compatibility effect (Kunde, 2001, Exp. 1), participants responded slower when producing incompatible rather than compatible action effects. This observation is consistent with the idea that effect anticipations are functionally relevant for human action control.

The effect size observed in the present sample was clearly smaller than the originally published effect size (*d*_*z*_ = 0.33 as compared to *d*_*z*_ = 0.96).^6^ Observing a small effect for this design also seems to be plausible, because action effects were a mere by-product of the response and thus entirely task-irrelevant. We would, therefore, assume the small sample size of the original study to have yielded an inflated effect size estimate. This reading of the data is also in line with the observation that the confidence interval around the original effect size estimate spans a considerable range, including the present estimate. It thus seems to be useful to conceptualize R-E compatibility effects as being of small size if the action-effect mapping is not relevant to the task at hand. Follow-up research with this approach has further suggested that task-relevance boosts R-E compatibility effects (Ansorge, 2002; Janczyk et al., 2015; Zwosta et al., 2013). The present observation of a small effect size should thus be seen as applying specifically to settings in which action effects are not relevant to the task at hand. Settings with task-irrelevant effects, however, might be regarded as providing particularly strong evidence for the functional role of effect anticipations if they still yield a reliable impact of R-E compatibility. We, therefore, believe that the present estimate provides useful information for future studies that aim to delineate when human action control draws on environment-related action effects in addition to or even instead of body-related action effects such as proprioceptive and kinaesthetic reafferences triggered by the moving body (Pfister, 2019; Pfister et al., 2014c; Thébault et al., 2018; Wirth et al., 2016).

Owing to the higher power of the present study relative to the original one, the data also suggest a reliable impact of the order of R-E mappings that had not been significant in the original study (Kunde, 2001). Responses were faster when participants began with the incompatible R-E mapping. This observation might suggest that participants did not include the experimentally induced action effects into their action representations when starting with an incompatible mapping so that they could establish efficient stimulus–response associations between target colors and corresponding body-related action effects. When transitioning to the compatible condition, they might have consolidated these associations sufficiently strong so that they established stimulus-effect associations independently of the existing stimulus–response associations. When starting with the compatible mapping, in contrast, they might have been inclined to integrate stimuli, responses, and effects in joint associations instead. This explanation is of course speculative at this point. Its speculative nature highlights that the interplay of pre-existing and newly learned R-E mappings is poorly understood at present which requires additional work to establish when which kinds of action effects are preferably integrated into action representations, and whether different R-E associations can be built up and retrieved in parallel.

A final consideration pertains to different types of R-E compatibility. Even though the present design with spatial compatibility between keypress responses and discrete visual effects likely is the most common version used (e.g., Ansorge, 2002; Janczyk & Lerche, 2019; Janczyk et al., 2017; Pfister & Kunde, 2013; Pfister et al., 2010; Shin & Proctor, 2012), there are numerous other viable possibilities to implement dimensional overlap between responses and effects (Kornblum et al., 1990). R-E compatibility effects were also reported and discussed, for example, with overlap on the dimensions intensity (Kunde, 2001; Kunde et al., 2004), duration (Kunde, 2003; Pfister et al., 2013), as well as for semantic compatibility (Földes et al., 2018; Hubbard et al., 2011; Koch & Kunde, 2002; Koch et al., 2021). Different response modalities included tool-transformed movements such as rotations via steering wheels and flight yokes (Janczyk et al., 2012c, 2015; Yamaguchi & Proctor, 2011), and the operation of one-pivot levers (Janczyk et al., 2012b; Kunde et al., 2007, 2012), as well as continuous mouse movements (Pfister et al., 2014b; Hommel et al., 2017; Wirth et al., 2015; but see Schonard et al., 2021). One case where no R-E compatibility effect was observed concerned touchless gestures (Janczyk et al., 2019). In general though, each of these studies set out to test specific theoretical predictions or applied scenarios that go beyond the scope of this investigation. They also yielded highly different effect size estimates, with many studies exceeding the effect size reported here. When approaching a novel setting without direct precursors in the literature, it seems useful to plan for the possibility of R-E compatibility effects being relatively small, thus requiring sufficiently many participants for meaningful investigations.

## Experiment 2: action-induced blindness

Perhaps one of the most counterintuitive implications of TEC is that already the planning of an action can have a direct impact on perception. This should occur if one particular feature (e.g., “left”) is part of an event file representing an action plan already (Hommel et al., 2001). If a perceptual event then requires the same feature (e.g., identifying a left visual impression), discrimination should be worse compared to a situation in which a different feature is required for the perceptual event (e.g., identifying a right visual impression).

**Fig. 4:**
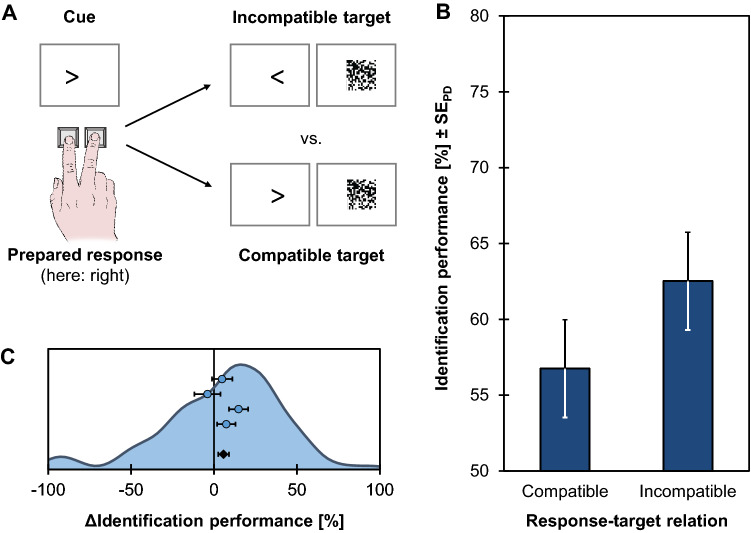
Design and results of the action-induced blindness experiment (Exp. 2). **A** Participants prepared a left or right keypress response as indicated by an arrow cue. While holding this response active, but before actually executing it, they were briefly presented with a masked arrow target. The main variable of interest is the percentage of correct target identifications (identification performance) as a function of whether the target arrow was compatible or incompatible to the prepared response. **B** Identification performance as a function of response-target compatibility. Error bars indicate standard errors of paired differences (SE_PD_; Pfister & Janczyk, 2013). **C** Distribution of individual action-induced blindness effects (ΔIdentification performance, computed as the difference between the compatible and incompatible condition; shown as kernel density estimate) together with means and standard errors of the four samples (blue dots) and the pooled data (black diamond)

This implication has been studied most prominently in action-induced blindness experiments, and Experiment 2 of our study replicates the original observation of this effect (Exp. 1 of Müsseler & Hommel, 1997a). In this design, participants were to plan a left or right response indicated by an arrow cue as shown in Fig. 4A. While keeping this action plan active, and before actually executing the action, participants were briefly presented with another arrow that either could match the spatial feature of the preceding cue and the prepared response or could point into the opposite direction. This target arrow was masked, and participants were asked to identify its direction and report this later with a key press. They then carried out the planned response and reported the identified target direction at the end of the trial. The main question was whether identification performance would depend on the overlap between the planned response and the target arrow.

### Method

#### Responses and stimuli

Responses were given with the right index and middle finger on the ‘g’ and ‘h’ key. Stimuli were left and right arrows (‘ < ’ and ‘ > ’), presented in white against a black background. Masks were 40 × 40 px squares in which 50% of randomly determined pixels were colored white.

#### Task and procedure

The experiment consisted of a pretest phase and a test phase. During the *pretest phase*, the stimulus presentation time was individually determined to achieve a detection accuracy of 60–90%. In each trial, the arrow stimulus was presented when participants pressed both response keys simultaneously and was briefly thereafter covered by a mask. The participants’ task was to press the left or right response key according to arrow direction. A short beep (500 Hz, 50 ms) indicated an error. Eight blocks of 12 trials were administered, consisting of six repetitions of both arrow directions. Initially, the arrows were presented for 70 ms. When detection accuracy exceeded 90% (i.e., ≤ 1 error per block), the presentation time was reduced by 14 ms (with a minimum of 14 ms), and when detection accuracy was below 60% (i.e., ≥ 5 errors), the presentation time was increased by 14 ms. The final presentation times ranged from 14 to 112 ms (14 ms: 9 participants; 28 ms: 53 participants; 42 ms: 18 participants; 56 ms: 19 participants; 70 ms: 13 participants; 84 ms: 6 participants; 98 ms: 1 participant; 112 ms: 1 participant).

In the subsequent *test phase*, participants had to (1) prepare one response (R1) to an initially presented arrow (S1; “response cue”) and (2) to identify a briefly presented and masked arrow (S2) and indicate its direction with a second response (R2). A trial began with the presentation of S1 (504 ms) slightly to the left of the screen center, followed by a blank screen. The trial commenced, when both keys were pressed simultaneously (R0), and S2 was presented slightly to the right of the screen center for the previously determined presentation time, after which it was masked. Participants then were to give the speeded R1 within 1000 ms. Otherwise, the trial was aborted. The mask remained on screen for another 1008 ms, and its offset signaled to the participants to give the (unspeeded) R2. In case of any errors, respective error feedback was provided (500 ms) at the end of a trial together with a short beep (500 Hz, 50 ms). The next trial started with an ITI of 500 ms. The test phase comprised 16 blocks with twelve trials each, resulting from three repetitions of 2 S1 × 2 S2 combinations.

#### Design and analyses

All participants were included into the analyses. Trials with unspecific errors (R0 during presentation of S1, R1 not within 1000 ms) were excluded first. RT0 was measured from S1 onset until R0, RT1 was measured as the interval between R0 and R1, and RT2 was measured from mask offset until R2.

The independent variable of interest was compatibility (compatible: S1 and S2 into the same direction vs. incompatible: S1 and S2 into opposite directions) as a repeated-measure. Mean (correct) RT0, RT1, and RT2, percentages of errors for R1, and percentage correct (PC) for R2 (i.e., identification performance) were analyzed with paired *t*-tests (one- or two-tailed as in the original analyses in Müsseler & Hommel, 1997a, 1997b). Individual action-induced blindness effects were calculated as ΔIdentification performance = PC2_incompatible_ – PC2_compatible_.

## Results

Errors in R1 were made in 6.09% of the compatible trials and in 13.23% of the incompatible trials, *t*(119) = 5.10, *p* < .001, *d* = 0.47 (two-tailed).

The percentage of correctly identified arrows compatible with the prepared R1 was 56.75 and it was 62.62 when the arrow and R1 were incompatible (see Fig. 4B, C), *t*(119) = 1.79, *p* = .038, *d* = 0.16 (one-tailed), 95% CI_SM_ = [− 0.02, 0.34].

The double key press was initiated on average 1556 ms after S1 onset. Mean RT1s were 471 ms for both the compatible and the incompatible conditions, *t*(119) = 0.15, *p* = .882, *d* = 0.01, and mean RT2s were 639 ms for both the compatible and the incompatible condition, *t*(119) = 0.01, *p* = .996, *d* < 0.01.

### Discussion

Targets were indeed identified less accurately when they faced in the direction of a planned key press (i.e., when the prepared key press and arrow direction were compatible) than when they faced in the opposite direction (i.e., when prepared key press and arrow direction were incompatible). However, the effect was considerably smaller (*d* = 0.16) than the medium-sized effect in the original study (*d* = 0.61).

Similar results were reported by several other studies using spatial compatibility (Müsseler & Hommel, 1997b; Müsseler et al., 2001; Thomaschke et al., 2018) as well as compatibility between pronounced color words and visually presented color patches (Kunde & Wühr, 2004). Going beyond intrinsic overlap of action features and events in the environment, action-induced blindness has also been observed for newly induced action effects such as specific onset and offset events in a visual display (albeit using the somewhat different measure of identification speed rather than accuracy; Pfister et al., 2012).

It is an open question, however, how these blindness effects relate to attenuated sensory processing after a response has actually been carried out. Findings of sensory attenuation following own actions were reported for neurophysiological measures such as event-related potentials (Horváth, 2015; Klaffehn et al., 2019; Timm et al., 2014; see also Schafer & Marcus, 1973). Corresponding behavioral findings have been reported only rarely in the literature, and if such effects were observed, they also tended to be of small size for body-external action effects (Weiss et al., 2011a, 2011b; for attenuated perception of body-related effects, see Bays et al., 2005, 2006; Shergill et al., 2003). It is tempting to attribute action-induced blindness during action planning and sensory attenuation after action execution to a single mechanism, that is, integration of feature codes into an event file (but see Thomaschke, 2012; Thomaschke et al., 2012). Additional work at the intersection of both phenomena is thus well-advised to recruit sufficiently large sample sizes, especially when addressing behavioral proxies of sensory processing.

A first relevant distinction between action-induced blindness and sensory attenuation is the question of how specific both effects are relative to different perceptual events. Here, action-induced blindness operates on precisely those feature codes that are represented as part of an action plan (Müsseler & Wühr, 2002). The blindness effect, therefore, applies only to a clearly defined subset of incoming features, whereas most empirical demonstrations of sensory attenuation can also be explained by nonspecific mechanisms (Horváth, 2015). For instance, focusing on planning and controlling a movement could be assumed to interfere with the processing of task-irrelevant sensory information, such as action-triggered changes in the environment (Horváth et al., 2012). Only attenuation effects that apply to specific features as represented in an action plan can, therefore, be assumed to derive from a similar mechanism as action-induced blindness. Because control conditions to assess such specificity have rarely been included in experiments on sensory attenuation, however, answering this question will require a larger empirical database. A second potentially relevant distinction between action-induced blindness and sensory attenuation pertains to the modality of to-be-perceived events. Whereas blindness effects were obviously probed in the visual modality, sensory attenuation has not yet been shown for behavioral identification of visual stimuli (Schwarz et al., 2018a). These circumstances might also relate to small population effects. TEC’s mechanism of feature integration is not tied to a specific modality, however, so that it would be interesting to investigate conceptually similar effects to action-induced blindness also in other modalities that have previously yielded robust effects of sensory attenuation (i.e., auditory and tactile events).

## Experiment 3: response-effect learning

Performing a bodily movement as an action, that is, as a means for achieving a particular goal, requires that a particular motor movement becomes associated with its consequences at first. Only then, these associations can be used for intentionally generating a movement, for example, via recollecting and anticipating the desired goal states (see also the introduction to Exp. 1 of the present paper). Based on these insights, already expressed by early formulations of the ideomotor principle (Harleß, 1861; Herbart, 1825; Lotze, 1852), Elsner and Hommel (2001) suggested a two-stage model of action control with Stage 1 concerning the acquisition of movement-effect relations and Stage 2 concerning their use in the course of intentional action control.

**Fig. 5:**
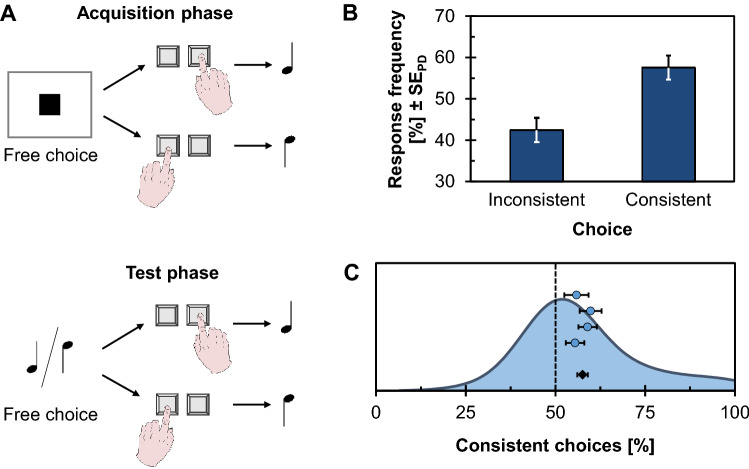
Design and results of the response-effect learning experiment (Exp. 3). **A** The experiment consisted of an acquisition phase followed by a test phase. In the acquisition phase, participants freely chose between pressing a left or a right key, and each key press triggered a contingent effect tone of distinct pitch. The test phase was similar except that the previous effect tones now also prompted each choice, whereas a third tone indicated a no-go trial to discourage response preparation ahead of the trial. **B** Response frequency for consistent and inconsistent choices. Error bars indicate standard errors of paired differences (SE_PD_; Pfister & Janczyk, 2013). **C** Distribution of individual percentages of consistent choices (shown as kernel density estimate) together with means and standard errors of the four samples (blue dots) and the pooled data (black diamond)

The experiments reported by Elsner and Hommel (2001) focused on Stage 1 and followed a general principle. During an initial acquisition phase, left versus right key presses were predictably followed by a low- or high-pitch tone. The repeated exposure to these contingencies was thought to induce (bidirectional) associations between responses and their effects. In contrast to, for example, R-E compatibility experiments, no dimensional overlap (Kornblum et al., 1990) is required and the combination of responses and effects could be arbitrary. In a subsequent test phase, the effect tones were then presented as stimuli and participants were either asked to make speeded forced-choice responses (Elsner & Hommel, 2001, Exp. 1) or free- choice responses to this tone stimulus (Exp. 2–4). In particular, our Experiment 3 replicates Experiment 3a of Elsner and Hommel, where participants were asked to freely choose between a left versus right key press upon hearing the low- or high-pitch tone (thus a free-choice task; Berlyne, 1957). Figure 5A shows a sketch of this procedure. If associations were learned, the associated response should receive some activation and a bias toward that response associated with the tone (i.e., as consistent response choice) is expected. To prevent participants from advance response selection, half of the trials in the test phase were no-go trials in which participants needed to refrain from responding. These trials were signaled by a bell chiming sound. This procedure thus ensured that activation by the previous action effect could indeed bias response choices.

### Method

#### Responses and stimuli

Responses were given with the left and right index finger on the left and right ‘ctrl’ keys. A white square presented centrally against a black background was used as a visual stimulus. Two sinusoidal tones (400 Hz [low tone] and 800 Hz [high tone], 200 ms) were used as auditory action effects. These tones were also used as go-stimuli in the test phase in addition to a bell chiming sound (~ 200 ms) as the no-go stimulus.

#### Task and procedure

The experiment consisted of an acquisition phase and a test phase. During the *acquisition phase*, the participants’ task was to respond with a left or right key press to a stimulus in a free-choice task. Each trial started with visual stimulus onset, and the stimulus remained on screen for 200 ms or until a response was given. If RTs were < 100 ms or > 1200 ms, respective written feedback was presented (500 ms), and the trial was repeated. In case of a valid response, an auditory action effect was played 50 ms after the response. For one half of the participants, the R-E mapping was left response → low tone and right response → high tone; for the other half of participants, this mapping was reversed. The next trial began after an ITI of 1500 ms. The acquisition phase began with 20 familiarization trials followed by four blocks of 50 valid trials each. Short breaks were allowed between these blocks and participants received written feedback about the number of left and right responses in the last block.^7^ Instructions asked the participants to choose the responses randomly and about equally often. The tones were described as being irrelevant.

The participants’ task in the *test phase* was to respond with a left or right key press to the low or high tone (go trials) and to refrain from responding when hearing the bell chiming sound (no-go trials). A trial began with the onset of the visual stimulus and the simultaneous presentation of the auditory stimulus. The visual stimulus remained on screen for 200 ms and a trial lasted 1200 ms maximally. If RTs in go trials were < 100 ms or > 1200 ms or a response was given in a no-go trial, respective written feedback was presented (500 ms). With 50 ms delay, correct responses in go trials were followed by an auditory action effect according to the mapping used in the acquisition phase. Invalid trials were repeated at a random position in the remainder of a block. The next trial started after an ITI of 1500 ms. Two test blocks of 100 valid trials were administered. In each block, 50% of the trials were no-go trials, and the two go stimuli were presented in 25% of the trials each. Instructions did not emphasize balanced choices, but participants were asked to avoid pressing only one of the response keys throughout.

### Design and analyses

One participant was excluded, because the experiment was cancelled after the acquisition block, and nine participants were excluded for responding in more than 10% of the no-go trials (in the original study, such participants were replaced with new ones).

Trials with RTs < 100 ms and RTs > 1200 ms were excluded first as anticipations and response omissions. RTs were measured from onset of the visual stimulus. The percentage of right responses during the acquisition phase was first calculated per participant and its deviation from chance was then assessed with a one-sample *t*-test.

The dependent variable of interest in the test phase was choice consistency in go trials. A choice in the test phase was consistent if the response and the auditory stimulus matched the combination from the acquisition phase; otherwise, it was inconsistent. For example, consider the R-E mapping in the acquisition phase where left response → low tone and right response → high tone. If a low tone was presented in the test phase, a left response would count as consistent, while a right response would count as inconsistent. The percentage of consistent choices was calculated per participant and tested against chance with a one-sample *t*-test. The percentage of consistent responses was used as the individual consistency effect. RTs of consistent and inconsistent choices were further compared with a paired *t*-test.

### Results

In the acquisition phase, participants committed 3.94% anticipations and 0.93% response omissions. Participants pressed the right key in 50.51% (SD = 2.26) of the trials, a slight but significant preference for this response option, *t*(109) = 2.39, *p* = .019, *d* = 0.23 (μ_0_ = 50).

In the test phase, anticipations did not occur, but 1.02% response omissions occurred, and participants responded in 2.43% of the no-go trials. Considering only valid (go) trials, participants chose a consistent response in 57.54% of the cases (see Fig. 5B), *t*(109) = 5.16, *p* < .001, *d* = 0.49, 95% CI_SM_ = [0.29, 0.69] (μ_0_ = 50). RTs were 671 ms and 675 ms for consistent and inconsistent choice, respectively, *t*(109) = 1.09, *p* = .276, *d* = 0.10.

#### Further analyses

Participants showed large differences in the percentage of consistent choices with a range from 25.00% to 99.00% as visible in the distribution of effects plotted in Fig. 5C. Values approaching either end of the scale could be seen as reflecting strategic rather than spontaneous responding. While such strategies were partially addressed in the original study by having participants count backwards in steps of three during the task (Elsner & Hommel, 2001, Exp. 4), it is currently unclear whether strategic responding actually affected results in other designs. Conceptually related studies have indeed suggested that several participants tend to apply such strategies (Dutzi & Hommel, 2009; Vogel et al., 2018; Weller et al., 2017). Dutzi and Hommel (2009) excluded participants with less than 10% consistent choices or more than 90% consistent choices as such patterns were argued to reflect strategies. In the present dataset, seven participants showed more than 90% consistent choices, suggesting a sizeable proportion of strategy users. We, therefore, reran the previous analysis with this subsample, additionally applying the more lenient criterion of excluding participants with more than 20% (instead of 10%) false alarms as done by Dutzi and Hommel (2009), which did not alter the results substantially, however.

The selected subsample of participants pressed the right key in 50.38% (SD = 2.58) of the trials, a non-significant preference for this response option, *t*(110) = 1.56, *p* = .121, *d* = 0.15 (μ_0_ = 50). The frequency of consistent response choices was slightly reduced to 54.70% of the trials, but still greater than expected by chance, *t*(110) = 4.34, *p* < .001, *d* = 0.41 (μ_0_ = 50). RTs were 666 ms and 670 ms for consistent and inconsistent choices, respectively, *t*(110) = 1.37, *p* = .172, *d* = 0.13.

### Discussion

This experiment replicated the results obtained by Elsner and Hommel (2001, Exp. 3a). Participants showed a bias toward consistent response choices, as would be expected if (bidirectional) associations between responses and effects have been established during the acquisition phase. The effect size (*d* = 0.49) was about half of the original effect size (*d* = 0.94).

While this experiment (as well as Elsner & Hommel, 2001, Exp. 2–4) employed a free-choice test phase and focused on choice rates, other experiments focused on RTs and used a forced-choice test phase in which a particular response was demanded by the stimulus (e.g., Eder & Dignath, 2017; Elsner & Hommel, 2001, Exp. 1; Hoffmann et al., 2009, Exp. 1; Hommel, 1996; Hommel et al., 2003). Responses in such experiments were faster when the stimulus was previously presented as the effect of the required response. Similar results were even obtained with subliminally presented stimuli (Kunde, 2004). In addition, Wolfensteller and Ruge (2011) investigated the time-course of R-E learning and systematically varied the amount of acquisition trials (200 in Elsner & Hommel, 2001). Reliable effects in RTs were obtained after only eight repetitions of R-E episodes already. Finally, a discussion, albeit one going beyond the present paper, has evolved around whether R-E learning occurs only with free-choice acquisition phases (Herwig & Waszak, 2009; Herwig et al., 2007) or also with forced-choice acquisition phases (Pfister et al., 2011).

A particularly relevant question for future work is whether R-E learning generalizes to settings that are more complex than the typical focus on two simple and distinct responses with perfectly contingent effects. Such situations therefore comprise multiple action opportunities (Watson et al., 2015), imperfect R-E contingencies (Elsner & Hommel, 2004), as well as actions that unfold over extended timescales (Land, 2018). Extending the available database on this question might also prove useful for implementing computational approaches to ideomotor learning (Herbort & Butz, 2012).

## Experiment 4: stimulus–response binding

Once response and stimulus features are integrated into an event file, TEC assumes that these features are not available for other event files. If the same situation occurs again, the response that is included in the preceding event file is retrieved and initiated swiftly. If a situation requires only stimulus and response features that are not currently part of an event file, the cognitive system can establish a new event file seamlessly as well. Issues arise, however, if required stimulus and response features partly overlap with an existing event file, thus yielding partial repetition costs whenever stimuli and responses partially match an immediately preceding response, as compared to complete repetitions or full alternations of an S-R episode (Hommel, 2004).

**Fig. 6:**
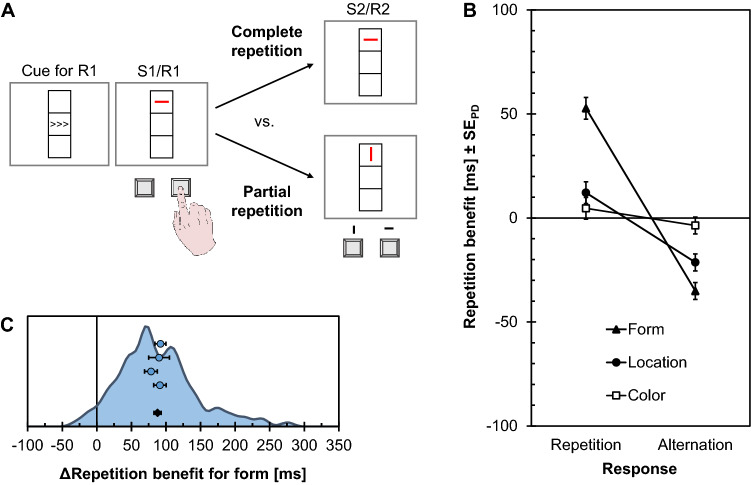
Design and results of the stimulus–response binding experiment (Exp. 4). **A** Participants prepared a first response (R1) as indicated by a cue and they executed this response upon the onset of a colored line that was oriented either horizontally or vertically (S1). A second line stimulus followed shortly thereafter, prompting a response depending on line orientation (S2). Trials were constructed so that all possible response and stimulus sequences were varied orthogonally, allowing to assess the integration of stimulus and response features into event files via partial repetition costs (computed as the difference in repetition benefits for features between response repetitions and response alternations; see text for details). **B** Stimulus feature repetition benefits (positive) and costs (negative) calculated as RT_alternation_ − RT_repetition_ for stimulus form, color, and location, as a function of response sequence (repetition vs. alternation). RT_alternation_ corresponds to the mean of all conditions involving an alternation of the respective stimulus feature while RT_repetition_ corresponds to the mean of all conditions with repetition of that feature. Error bars indicate standard errors of paired differences (SE_PD_; Pfister & Janczyk, 2013). **C** Distribution of partial repetition costs (ΔRepetition benefit) for the task-relevant stimulus feature form (shown as kernel density estimate) with means and standard errors of the four samples (blue dots) and the pooled data (black diamond)

An experimental approach to demonstrate such partial repetition costs are S1R1-S2R2 experiments as shown in Fig. 6A, which was implemented in Experiment 4 as a replication of Experiment 1b of Hommel (1998). Participants responded with a prepared left or right response upon encountering a stimulus with three variable features (form, location, color). Following this, another stimulus was presented and required a response to its form. Of particular interest was whether a benefit of repeating the response depended on simultaneous repetitions of stimulus features. Binding of a response and a stimulus feature into the same event file was assumed if benefits of response repetitions (compared to response changes) were more pronounced for simultaneous stimulus feature repetition than for stimulus feature change. This observation has been taken to extend the notion of perceptual *object files* (Kahneman & Treisman, 1984; Kahneman et al. 1992) to *event files* that also include active responses (Hommel, 1998, 2004; see also Henderson, 1996).

### Method

#### Responses and stimuli

Responses were given with the left and right index finger on the left and right ‘ctrl’ keys (note that responses were given with the right index and middle finger in the original study). A vertical stack of three square-shaped, white outlines was presented against a black background throughout the experiment. White arrows (‘ <  <  < ’ or ‘ >  >  > ’) appeared in the middle square and served as response cues, whereas a red or green horizontal or vertical line appeared either in the top or the bottom square and served as S1 and S2.

#### Task and procedure

Participants were to prepare a left or right response according to the response cue, which was then given upon onset of S1. After this response, S2 occurred and participants were to give a left or right response to the form of S2 (i.e., whether the line was horizontal or vertical).

A trial began with the presentation of the response cue (1500 ms), which was always presented in the central square and which disappeared for 1000 ms. Then, S1 appeared in the upper or lower square (500 ms) and prompted the participant to give the prepared response R1, irrespective of the form, location, or color of this stimulus. If R1 was not given within 1000 ms after S1 onset or if R1 was not correct, respective error feedback was provided (500 ms) and the trial was aborted. Otherwise, S2 was presented in the upper or in the lower square until response R2 was given with a time limit of 2000 ms. Error feedback (500 ms) was provided in case of a late R2, if R2 was given prior to S2 onset, or if R2 was incorrect. The next trial started after an ITI of 2000 ms. Trials with errors were repeated at a random position in the remainder of the block.

The experiment started with a practice block of 40 randomly drawn valid trials (taken from the 128 possible trials, as described next). Then, two experimental blocks with 128 valid trials were administered. The 128 trials resulted from combining 2 response cues (‘ <  <  < ’ vs. ‘ >  >  > ’) × 2 S1 form (horizontal vs. vertical) × 2 S1 color (red vs. green) × 2 S1 location (upper vs. lower square) × 2 S2 form (horizontal vs. vertical) × 2 S2 color (red vs. green) × 2 S2 location (upper vs. lower square). Instructions emphasized speed and accuracy of responses.

#### Design and analyses

Three participants were excluded from the analyses, because they aborted the experiment and thus data were lacking. RT analyses were based on correct trials, while error analyses were based on the initial trials that were not added to a block due to errors. RT1s were measured from S1 onset until R1 and RT2s were measured from S2 onset until R2.

Trials were coded with regard to response sequence (i.e., R2 either repeated or alternated relative to the cued R1) and sequences of the stimulus features form (horizontal vs. vertical), location (upper vs. lower square), and color (red vs. green). Thus, the following four independent variables (all repeated-measures) were of interest: Response sequence (repetition vs. alternation), form sequence (repetition vs. alternation), location sequence (repetition vs. alternation), and color sequence (repetition vs. alternation).

Mean correct RTs and PEs in R2 were analyzed with a 2 × 2 × 2 × 2 ANOVA with response sequence, form sequence, location sequence, and color sequence as repeated-measures. Benefits and costs of stimulus feature repetitions were calculated as RT_alternation_—RT_repetition_ separately for response repetitions and alternations. Positive values for the difference reflect benefits, negative values reflect costs, and both were evaluated with one-sample *t*-tests against μ_0_ = 0. Partial repetition costs were assessed as the difference between the effects of stimulus feature sequences between response repetitions and response alternations. We mainly focused on the partial repetition costs for the task-relevant feature form, because this feature had yielded the largest effect in the original study (*d*_*z*_ = 2.70; Hommel, 1998). These costs were computed as ΔRepetition benefit = (RT_form alternation AND response repetition_ — RT_form repetition AND response repetition_) – (RT_form alternation AND response alternation_—RT_form repetition AND response alternation_). Partial repetition costs for the task-irrelevant features color and location were computed analogously.

### Results

R1s were incorrect, missing, or anticipated (RT1 < 100 ms) in 1.13%, 1.93%, and 3.52% of the trials. Mean correct RT1 was 413 ms. R2s were missing or anticipated in 0.90% and 0.13% of the trials, and these trials were excluded from further analyses.

#### Response times

Mean RTs for the different conditions of repeated stimulus features and responses are provided in Table 3, and details on the inferential statistics from the 4-way ANOVA are summarized in Table 4. Of most interest was whether a benefit of repeating stimulus form, color, or location depended on the repetition versus alternation of the response. Figure 6B visualizes the benefits (and costs, if negative) of repeating the stimulus features separately for response repetitions and alternations. Statistically, the interesting pattern translates into the two-way interactions of stimulus form, location, and color with response repetition, which were significant in all three cases (see also Fig. 6B). The corresponding partial repetition costs amounted to 88 ms for form, *d*_*z*_ = 1.56, 95% CI_SM_ = [1.28, 1.82] (see Fig. 6C for the distribution across participants), 34 ms for location, *d*_*z*_ = 0.76, 95% CI_SM_ = [0.56, 0.97], and 8 ms for color, *d*_*z*_ = 0.22, 95% CI_SM_ = [0.04, 0.41]. For stimulus form, both the benefits, *t*(116) = 16.83, *p* < .001, *d* = 1.56, and the costs were significant, *t*(116) = -9.73, *p* < .001, *d* = − 0.90. The same were true for stimulus location, benefits: *t*(116) = 4.37, *p* < .001, *d* = 0.40; costs: *t*(116) = − 6.23, *p* < .001, *d* = – 0.58. For stimulus color, however, only the benefits were significant, *t*(116) = 2.03, *p* = .044, *d* = 0.19, while the costs were not significant, *t*(116) = – 1.52, *p* = .131, *d* = – 0.14 (one-sample *t*-tests against μ_0_ = 0).

**Table 3:** Mean response times (RT; in ms) and percentages of errors (PE) for R2 in Experiment 4 as a function of the relationship between S1 and S2 and between R1 and R2 (R2 Repeated or Alternated)

	Response			
	Repeated		Alternated	
Stimulus feature repeated	RT	PE	RT	PE
Neither	584	9.28	536	1.63
F(orm)	553	4.75	582	7.53
L(ocation)	582	8.06	564	5.06
C(olor)	582	11.36	543	1.76
FL	527	1.70	599	14.63
FC	541	3.02	585	8.64
LC	594	8.91	575	5.65
FLC	509	2.11	593	16.08

**Table 4:** Inferential statistics from the four-way ANOVA on response times (RT) and percentages of errors (PE) of R2 of Experiment 4

	**RT**			**PE**		
**Effect**	*F***(1, 116)**	*p*	**η**_p_^2^	*F***(1, 116)**	*p*	**η**_p_^2^
Form	16.78	< .001	.13	4.40	.038	.04
Color	0.10	.750	< .01	4.29	.041	.04
Location	3.76	.055	.03	39.18	<.001	.25
Response	6.41	.013	.05	11.64	.001	.09
Form × Color	20.18	< .001	.15	1.39	.241	.01
Form × Location	60.49	< .001	.34	7.16	.009	.06
Color × Location	0.06	.809	< .01	0.50	.480	< .01
Form × Response	283.40	< .001	.71	180.89	< .001	.61
Color × Response	5.89	.017	.05	0.51	.476	< .01
Location × Response	68.26	< .001	.37	78.72	< .001	.40
Form × Color × Location	4.72	.032	.04	2.32	.131	.02
Form × Color × Response	1.39	.242	.01	6.73	.011	.06
Form × Location × Response	4.93	.028	.04	8.35	.005	.07
Color × Location × Response	0.77	.381	.01	< 0.01	.965	< .01
Form × Color × Location × Response	0.24	.623	< .01	2.35	.128	.02

#### Percentages error

Mean PEs for the different conditions of repeated stimulus features and responses are provided in Table 3, and details on the inferential statistics from the 4-way ANOVA are summarized in Table 4. The two-way interactions with response repetitions were significant for stimulus form and location, but not for stimulus color.

### Discussion

The integration of stimulus and response features into event files is at the heart of TEC, and Experiment 4 mirrors earlier reports in showing that re-encountering previous stimulus features retrieves event files, and thus corresponding responses (Hommel, 1998, 2004). Albeit smaller than in the original study, this setup still resulted in a considerably large effect size for task-relevant information (*d*_*z*_ = 1.56 as compared to *d*_*z*_ = 2.70 in the original). The present observations further mirror the original results in showing largest partial repetition costs for task-relevant stimulus features (here: form), and smaller partial repetition costs for task-irrelevant features (here: location and color; *d*_*z*_ ≤ 0.76). Notably, this pattern should indeed be seen as reflecting task-relevance rather than intrinsic properties of different features, because the original publication had already included a control experiment that showed a reversed pattern when color rather than location was implemented as task-relevant (Hommel, 1998, Exp. 2).

In contrast to the original data, however, the present results also yielded small partial repetition costs for the task-irrelevant feature of color. Because the corresponding interaction of color sequence and response sequence had not been significant in the original study, this study concluded (Hommel, 1998, p. 200): “Yet, the indication that colour information is not integrated with information about stimulus or response location suggests that feature binding is selective." The present data suggest that at least the design of the S1R1-S2R2 task rather supports the assumption of unselective binding, a notion that is reinforced by numerous replications of binding between task-irrelevant distractors and responses (Frings et al., 2007; Hommel, 2005; Moeller et al., 2016). Whereas binding seems to be effective by default, the amount of attention devoted to task-irrelevant information certainly affects retrieval of event files, however (Hommel et al., 2014; Moeller & Frings, 2014).

Another noteworthy observation was the fact that several three-way interactions were significant in our study (Form × Color × Location only for RTs, and Form × Color × Response only for PEs). This was not the case in the original study by Hommel (1998), and was interpreted as evidence for local and independent bindings between two features within the event file (see also Hommel, 2007). Similarly, results reported by Giesen and Rothermund (2014, 2016) also argue for independent, local, and binary bindings between relevant and irrelevant stimulus as well as response features within an event file. The present results challenge these conclusions though, albeit it is fair to say that the obtained three-way interaction effects range in the realm of small effect sizes only. This could certainly explain why higher-order interactions were not observed in previous studies with smaller sample sizes. Interestingly, the alternative view, that is, a “uniform-event file hypothesis” (Hommel, 1998, p.188) is also not fully supported by the present data, since the four-way interaction was not significant. Future research should systematically address issues relating to the structure of event files in more detail. Yet, since the higher order interactions seem to come with smaller effect sizes, such research would be well advised to plan sample sizes accordingly.

## Experiment 5: Code occupation

Experiments on action-induced blindness demonstrated that planning an action binds particular features that are consequently less available for perception of events requiring these features (e.g., Müsseler & Hommel, 1997a; see also Exp. 2 of the present paper). For example, planning a left response impaired perception of a left-oriented (compatible) arrow in comparison to a right-oriented (incompatible) arrow. Stoet and Hommel (1999) reported experiments extending this logic to two actions performed in succession. In other words, execution of an action was impaired if this action shared features with another action plan that was held active in working memory.

**Fig. 7:**
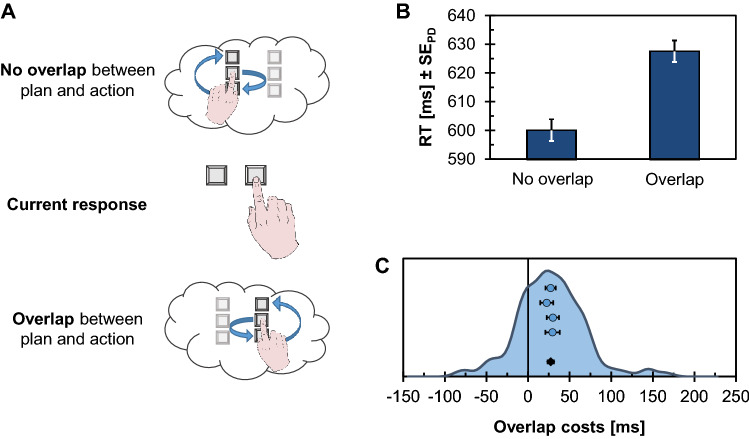
Design and results of the code occupation experiment (Exp. 5). **A** Participants prepared a response as indicated by a cue (*R*_A_) and they executed another response (*R*_B_) while holding the first response in working memory. Response times (RT_B_s) of this latter response were assessed as a function of whether this response overlapped or did not overlap with the action plan held in working memory to probe for occupation of the corresponding feature. **B** RT_B_s as a function of feature overlap between the current response and the action plan held in working memory. Error bars indicate standard errors of paired differences (SE_PD_; Pfister & Janczyk, 2013). **C** Distribution of overlap costs (shown as kernel density estimate) together with means and standard errors of the four samples (blue dots) and the pooled data (black diamond)

This experiment replicates Experiment 1 of Stoet and Hommel (1999). As sketched in Fig. 7A, participants prepared a response with the left or right index finger and were then required to give a speeded left or right response to a different stimulus, while the prepared response had to be held in working memory and was executed only thereafter. If planning a response binds a feature into an event file, performing an action on the same side (overlap) should be impaired compared to an action performed on the other side (no overlap).

### Method

#### Responses and stimuli

Responses were given with the left index finger on the keys ‘1’, ‘4’, and ‘7’ of the number pad, and with the right index finger on the keys ‘3’, ‘6’, and ‘9’ of the number pad. Visual stimuli were a white left or right arrow (‘ < ’ vs. ‘ > ’) presented at the screen center, the white numbers ‘1’ and ‘2’ presented below or above screen center (together forming S_A_, see below), and a red or green square presented at screen center (S_B_). All stimuli were presented against a black background.

#### Task and procedure

The participants’ task was to prepare a response R_A_ according to a stimulus S_A_. Then, a stimulus S_B_ appeared and required a speeded response R_B_. After this, participants executed the prepared *R*_A_.

A trial began when participants depressed the ‘4’ and the ‘6’ keys (‘home buttons’) with the presentation of a white fixation mark (‘*’, 350 ms). Then, S_A_ set on and remained on screen for 2000 ms. S_A_ consisted of an arrow that indicated whether the left or the right index finger was to be used for R_A_. Simultaneously, either only the number ‘1’ appeared below or above the arrow (‘easy condition’), or additionally the number ‘2’ appeared in the other position above or below the arrow (‘complex condition’). In the easy condition, R_A_ required releasing the home button (according to the arrow direction), pressing the respective key above (i.e., ‘7’ or ‘9’) if the ‘1’ appeared above the arrow, or below (i.e., ‘1’ or ‘3’) if the ‘1’ appeared below the arrow, and then return and press the home button again. In the complex condition, R_A_ became longer, and participants were to press a second key (in the direction of the ‘2’ above or below the arrow) following the first key before returning to the home button. Importantly, R_A_ was not yet be executed but only prepared. Following a blank screen (1000 ms), the white outline of a central square appeared for 350 ms. This square was then filled red or green (S_B_) and participants were to give a speeded response to this color by briefly lifting the left or right home button (R_B_). The prepared R_A_ was executed immediately after R_B_. A trial was aborted when not both responses were given within 5000 ms after onset of S_B_ or if R_B_ was incorrect. The next trial started when both home buttons were depressed again. Trials with errors were repeated at a random position in the remainder of the experiment.

The experiment started with a practice block of 32 valid trials (2 repetitions of 16 trial types) followed by a single experimental block with 320 valid trials (20 repetitions of 16 trial types). The 16 different trial types resulted from combining 2 S_B_ colors (red vs. green) × 2 S_A_ locations (left vs. right arrow) × 2 S_A_ complexities (easy [1-step response] vs. complex [2-step response]) × 2 S_A_ directions ([first] movement up vs. down). Instructions emphasized speed and accuracy of responses.

#### Design and analyses

Two participants were excluded from analyses, because they aborted the experiment and thus data were lacking.

RT analyses were based on correct trials, while error analyses were based on the initial trials that were not added to the experiment due to errors. RT_B_s were measured from S_B_ onset until the first release of the respective home button, and movement times in R_B_ (MT_B_s) were measured from this lift until the subsequent depressing of this home button. Inter-response times (IRTs) were measured from the end of R_B_ until the next release of a home button as part of R_A_, and movement times for R_A_ (MT_A_s) were measured from this release until the home button was depressed again at the end of R_A_. PE_B_ reflects the percentage of incorrect R_B_s, and PE_A_ reflects the percentage of incorrect R_A_s following a correct R_B_. Trials were excluded if any of the time-based variables was below 50 ms. This exclusion was not made in the original study, but we deemed it useful to focus the analyses on trials that were performed as intended. This criterion did not change the qualitative pattern of results, however.

Trials were coded with regard to (spatial) overlap conditions between R_B_ and R_A_, that is, whether both responses were given on the same side (overlap) or not (no overlap). Thus, condition (overlap vs. no overlap) was the major independent variable of interest. All dependent measures were analyzed with a paired *t*-tests comparing overlap and no overlap conditions. In a further analysis, complexity of R_A_ (easy vs. complex) was considered additionally and dependent measures were then submitted to 2 × 2 ANOVAs with condition (overlap vs. no overlap) and complexity (easy vs. complex) as repeated-measures. Individual overlap effects were calculated as overlap costs = RT_B | overlap_ – RT_B | no overlap_.

### Results

Mean RT_B_s, MT_B_s, and PE_B_s as a function of overlap are summarized in Table 5. RT_B_s and MT_B_s were longer with overlap compared to without overlap, RT_B_s: *t*(117) = 7.33, *p* < .001, *d* = 0.68, 95% CI_SM_ = [0.47, 0.87] (see Fig. 7B, C); MT_B_s: *t*(117) = 6.02, *p* < .001, *d* = 0.55. The difference for PE_B_s was not significant, *t*(117) = 0.74, *p* = .458, *d* = 0.07.

**Table 5:** Mean correct response times (RT), movement times (MT), inter-response times (IRT; all in milliseconds), and percentages of errors (PE) for Responses B and A as a function of condition (overlap vs. no overlap). Standard deviations are provided in parentheses

	Response B			Response A		
Condition	RT_B_	MT_B_	PE_B_	IRT	MT_A_	PE_A_
Overlap	627 (148)	193 (78)	2.38 (3.78)	167 (74)	569 (146)	0.75 (1.67)
No overlap	600 (147)	180 (68)	2.55 (2.76)	185 (79)	597 (145)	0.72 (1.23)

Mean IRTs, MT_A_s, and PE_A_s as a function of overlap are summarized in Table 5. IRTs and MT_A_s were shorter with overlap compared to without overlap, IRTs: *t*(117) = 4.55, *p* < .001, *d* = 0.42; MT_A_s: *t*(117) = 8.61, *p* < .001, *d* = 0.79. The difference for PE_A_s was not significant, *t*(117) = – 0.16, *p* = .875, *d* = – 0.01.

### Further analyses

Mean RT_B_s, MT_B_s, and PE_B_s as a function of overlap and complexity of R_A_ are summarized in Table 6. The ANOVA on RT_B_ revealed only a significant main effect of overlap, *F*(1, 117) = 54.59, *p* < .001, $\eta_{{\text{p}}}^{2}$ = .32. Neither the main effect of complexity, *F*(1, 117) = 0.51, *p* = .478, $\eta_{{\text{p}}}^{2}$ < .01, nor the interaction were significant, *F*(1, 117) = 0.07, *p* = .797, $\eta_{{\text{p}}}^{2}$ < .01. For MT_B_s, the main effect of overlap was significant, *F*(1, 117) = 36.16, *p* < .001, $\eta_{{\text{p}}}^{2}$ = .24. MT_B_s were also longer with complex compared to easy conditions, *F*(1, 117) = 6.71, *p* = .011, $\eta_{{\text{p}}}^{2}$ = .05. In addition, the effect of overlap was larger with complex R_A_ (16 ms) than with easy R_A_ (12 ms), and the interaction was significant, *F*(1, 117) = 7.23, *p* = .008, $\eta_{{\text{p}}}^{2}$ = .06. For PE_B_s, no effect was significant, overlap: *F*(1, 117) = 0.56, *p* = .457, $\eta_{{\text{p}}}^{2}$ < .01; complexity: *F*(1, 117) = 0.38, *p* = .541, $\eta_{{\text{p}}}^{2}$ < .01; interaction: *F*(1, 117) = 0.76, *p* = .385, $\eta_{{\text{p}}}^{2}$ = .01.

**Table 6:** Mean correct response times (RT), movement times (MT), inter-response times (IRT; all in milliseconds), and percentages of errors (PE) for Responses B and A as a function of condition (overlap vs. no overlap) and complexity of Response A (easy = one target, complex = two targets). Standard deviations are provided in parentheses

		Response B			Response A		
Complexity	Condition	RT_B_	MT_B_	PE_B_	IRT	MT_A_	PE_A_
Easy	Overlap	628 (148)	191 (75)	2.37 (3.32)	167 (72)	420 (116)	0.94 (1.83)
	No overlap	601 (148)	179 (67)	2.66 (4.19)	182 (76)	448 (118)	1.10 (2.21)
Complex	Overlap	627 (150)	196 (82)	2.39 (2.78)	168 (177)	718 (177)	0.54 (2.09)
	No overlap	599 (148)	180 (69)	2.43 (3.72)	189 (175)	746 (175)	0.34 (0.86)

Mean IRTs, MT_A_s, and PE_A_s as a function of overlap and complexity of R_A_ are summarized in Table 6. For IRTs, only the main effect of overlap was significant, *F*(1, 117) = 20.68, *p* < .001, $\eta_{{\text{p}}}^{2}$ = .15. Neither the main effect of complexity, *F*(1, 117) = 2.07, *p* = .153, $\eta_{{\text{p}}}^{2}$ = .02, nor the interaction were significant, *F*(1, 117) = 3.08, *p* = .082, $\eta_{{\text{p}}}^{2}$ = .03. For MT_A_s, the main effect of overlap was significant, *F*(1, 117) = 75.72, *p* < .001, $\eta_{{\text{p}}}^{2}$ = .39, and, trivially, MT_A_s were longer in the complex than in the easy condition, *F*(1, 117) = 2427.78, *p* < .001, $\eta_{{\text{p}}}^{2}$ = .95. The interaction was not significant though, *F*(1, 117) = 0.01, *p* = .938, $\eta_{{\text{p}}}^{2}$ < .01. For PE_A_s, only the main effect of complexity was significant with more errors in easy than in complex trials, *F*(1, 117) = 12.07, *p* = .001, $\eta_{{\text{p}}}^{2}$ = .09. Neither the main effect of overlap, *F*(1, 117) = 0.03, *p* = .872, η_p_^2^ < .01, nor the interaction were significant, *F*(1, 117) = 2.70, *p* = .103, $\eta_{{\text{p}}}^{2}$ = .02.

### Discussion

As in the original study, responses were initiated more quickly when they did not overlap with an action plan held in memory as compared to situations with overlapping response sides (Stoet & Hommel, 1999). The effect size was, again, smaller for the present results (*d* = 0.68) compared with the original experiment (*d* = 1.01). In addition to significant effects for RT_B_, but in contrast to the original experiment, MT_B_s were also significantly different as were IRTs and MT_A_s. Thus, preparing a response appears to bind features that are subsequently less available for other responses. We also additionally included the factor complexity into analyses; yet, these analyses offered no further relevant insights.

Of particular theoretical interest in this kind of experiments is that features of an event file compete between multiple action plans as compared to a single action plan and unrelated perceptual processing. Feature binding can therefore affect the interplay of different actions, and can also bias the choice for one action alternative over another (Richardson et al., 2020). This interplay extends to situations that include even more complex, multi-step actions (e.g., Fournier & Gallimore, 2013). For example, it has been shown that partial overlap with the first response in a sequence A hinders response B execution more than partial overlap with a later part of the sequence (Fournier et al., 2014). Hence, different parts of complex actions seem to be bound into the event file to different degrees. Notably, this is not the case, if the bound responses were individually planned, rather than being part of one more complex action (Moeller & Frings, 2019a; Moeller & Frings, 2019b). The question how longer action sequences are cognitively represented and what the structure of such events looks like is an important one, as it might move theoretical implications from TEC to more natural everyday tasks (e.g., Vallacher & Wegner, 1987; Yamaguchi & Logan, 2014; Zacks & Tversky, 2001; Zacks et al., 2001) and vice versa (see Moeller & Frings, 2021). Yet, since this is still a relatively new area in the literature, much more research is needed, to achieve a clearer understanding of structures within such events and solve questions such as under what circumstances response order becomes part of the event representation. The effect size estimation of our Experiment 5 suggests that researchers can plan with a medium-sized effect in this area.

## Experiment 6: short-term response-effect binding

Most studies on R-E learning (e.g., Elsner & Hommel, 2001; see also Exp. 3 of the present paper) focused on acquisition of R-E associations after repeated exposures to such episodes. Wolfensteller and Ruge (2011) already suggested that only relatively few acquisition trials are required for building such associations. However, similar to how stimulus features and responses also become transiently integrated into an event file (Hommel, 1998; see Exp. 4 of the present paper), experiencing an episode of one response and its effect should yield an event file as well (see also Hommel, 2005). If briefly thereafter the effect feature is encountered again, retrieval of the whole event file is expected to induce a bias toward the integrated response. This idea is tested in experiments on short-term R-E binding (Dutzi & Hommel, 2009).

**Fig. 8:**
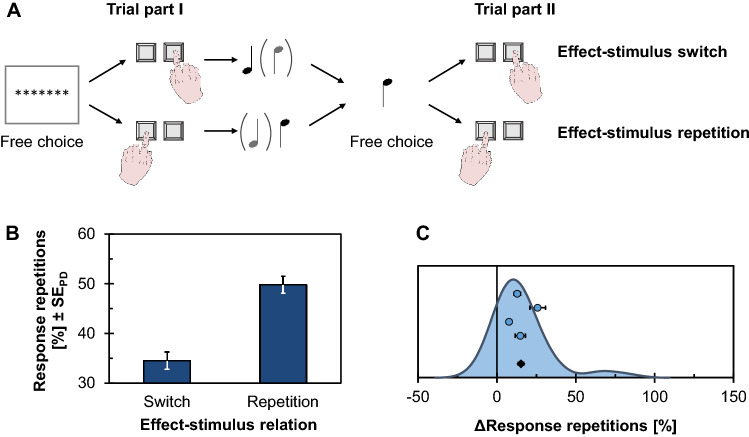
Design and results of the response-effect binding experiment (Exp. 6). **A** Each trial comprised two parts. In the first part, a freely chosen response triggered one of two possible effect tones at random. This effect tone was either repeated as the stimulus for the second part (effect-stimulus repetition) or the alternative tone was used as the stimulus (effect-stimulus switch). Short-term binding of response and following effects would be evident in higher response repetition rates across trial parts for effect-stimulus repetitions than for effect-stimulus switches. **B** Response repetitions as a function of effect-stimulus relation. Error bars indicate standard errors of paired differences (SE_PD_; Pfister & Janczyk, 2013). **C** Distribution of binding effects, measured as the difference in response repetitions between conditions (shown as kernel density estimate) together with means and standard errors of the four samples (blue dots) and the pooled data (black diamond)

The present experiment replicates Experiment 1 of Dutzi and Hommel (2009) and focused on short-term bindings from one to the next response. Participants’ free-choice responses resulted in one of two auditory action effects. In the majority of trials, either the same tone or the other tone was then presented as a stimulus requiring a second free-choice response as shown in Fig. 8A (effect-stimulus repetition vs. switch). If responses and effects are integrated into an event file and its retrieval affects response choice, a higher proportion of repeated responses is expected for effect-stimulus repetitions as compared to switches.

### Method

#### Responses and stimuli

Responses were given with the left and right index finger on the left and right ‘ctrl’ keys. A row of 13 white asterisks presented against a black background was used as a visual stimulus and two sinusoidal tones (400 Hz [low tone] and 800 Hz [high tone], 100 ms) were used as auditory effects and stimuli.

#### Task and procedure

The participants’ task was to first respond in a free-choice task to the visual stimulus. After an auditory effect to this response, another tone was played as a go stimulus for a second free-choice response. If no tone was played (i.e., in no-go trials), no response was to be given.

A trial began with the presentation of the visual stimulus (S1) for 300 ms asking for a speeded left or right response (R1). After R1 was given, either the low or high tone was played. The pitch of both tones was randomly distributed across a block of trials, with the restriction that both tones appeared equally often (note that tone type was determined randomly on each trial in the original study). Following another 1000 ms without key press (if participants pressed a key during this period, the trial was aborted), another tone (S2) was presented on 75% of the trials. In half of these trials, the tone following R1 was repeated (effect-stimulus repetition); in the other half, it switched, meaning that the other tone was played (effect-stimulus switch). In these go trials, participants were to give another free-choice left or right response (R2) within 1500 ms. In the remaining 25% of the trials, no S2 was presented, and participants had to refrain from pressing a key (no-go trial). Error feedback (1000 ms) was given if, in go trials, the response was faster than 100 ms or slower than 1500 ms, or if a response was given in no-go trials. The next trial started after an ITI of 3000 ms.

The experiment started with ten randomly drawn familiarization trials which were followed by three blocks of 64 trials each (16 no-go trials, 24 congruent go trials, 24 incongruent go trials). Instructions emphasized spontaneous and random responding in the free-choice R1 and R2 without using any strategies, and to respond as fast as possible.

#### Design and analyses

We excluded participants following the same criteria as applied by Dutzi and Hommel (2009, see p. 429). Five participants were excluded for more than 20% false alarms in no-go trials, six participants were excluded for response repetitions in less than 10% or more than 90% of the trials, and another six participants were excluded, because of less than 90% correct trials altogether. RT2 was measured from S2 onset (in go trials) until R2.

Trials were coded as to whether R2 was a repetition or a switch with respect to R1. The percentage of response repetitions was the main dependent variable in this experiment and was compared between effect-stimulus repetition and switch trials with a paired *t*-test. The difference between both conditions, ΔResponse repetitions, was used to quantify the binding and retrieval effect for each individual. RT2s were additionally analyzed with a paired *t*-test. The percentage of right responses as R1 was calculated per participant and then compared against chance with a one-sample *t*-test (μ_0_ = 50).

### Results

Based on RT2s, 0.96% of the trials were excluded as anticipations (RT2 < 100 ms) and 0.79% were excluded as response omissions (RT2 > 1500 ms).

Participants pressed the right key as R1 in 55.00% of the trials, a value significantly deviating from 50%, *t*(104) = 5.82, *p* < .001, *d* = 0.57. Response repetitions occurred in 49.72% of the effect-stimulus repetition trials and in 34.51% of the effect-stimulus switch trials. This difference was significant, *t*(104) = 8.76, *p* < .001, *d* = 0.86, 95% CI_SM_ = [0.63, 1.08] (see Fig. 8B, C).

RT2s in response alternations were shorter (561 ms) than in response repetitions (578 ms), *t*(104) = 5.02, *p* < .001, *d* = 0.49.

#### Further analyses

R2s in effect-stimulus repetition trials were slower (577 ms) than in effect-stimulus switch trials (557 ms), *t*(104) = 6.80, *p* < .001, *d* = 0.66.

### Discussion

The expected higher proportion of response repetitions in congruent compared with incongruent trials was observed and thus this experiment replicated Experiment 1 of Dutzi and Hommel (2009). In contrast to the previous experiments, the effect size (*d* = 0.86) was closer to the one reported in the original study, albeit still smaller (original *d* = 1.15). Similar results were obtained in several studies (e.g., Janczyk et al., 2012a; Schwarz et al., 2018b). Previous work further sought to investigate whether short-term action-effect binding is related to S-R binding on the one hand (Moeller et al., 2016, 2019), and to longer term R-E learning on the other hand (Herwig & Waszak, 2012). Especially the relation of short-term binding to stable long-term associations is still poorly understood, however, so that future studies can build on the present effect size estimate.

## General discussion

In the past two decades, TEC (Hommel et al., 2001) has become an influential framework for integrating the domains of perception and action in cognitive psychology. TEC has incorporated many empirical phenomena and has stimulated a vast amount of research with different experimental approaches since its first publication. A drawback of these earlier experiments is their use of rather small sample sizes yielding effect size estimates that likely overestimate the true effect.

Based on a survey of experts in the field, we selected six empirical phenomena rated as most relevant for TEC. The major goal of the study was to provide effect sizes of these phenomena with a 95% confidence interval of standardized means with sufficient precision (defined as width = 0.4). To this end, 120 participants performed in six experiments modelled closely after the original ones.

### Summary of results and their implications

The bottom line of the following results is straightforward: (1) All phenomena were replicated as in the original study. (2) The resulting effect sizes were (much) smaller though when compared with that reported in the original publications (see Table 7 and also Fig. 2 for a graphical summary).^8^

**Table 7:** Overview of effect sizes of the original studies and the corresponding new experiments reported in the present study, with 95% confidence interval for standardized means in brackets

		Effect size		Required sample size *n* for …	
Exp	Phenomenon	Original	New	1 − *β* = .8	1 − *β* = .9
1	Response-effect compatibility (Kunde, 2001)	0.96	0.33 [0.14, 0.51]	59/75	81/99
2	Action-induced blindness (Müsseler and Hommel (1997a, 1997b)	0.61	0.16 [−0.02, 0.34]	243/309	336/413
3	Response-effect learning (Elsner & Hommel, 2001)	0.94	0.49 [0.29, 0.69]	28/35	38/46
4	Stimulus–response binding (Hommel, 1998)	2.70	1.56 [1.28, 1.82]	5/6	6/7
5	Code occupation (Stoet & Hommel, 1999)	1.01	0.68 [0.47, 0.87]	15/19	20/25
6	Short-term response-effect binding (Dutzi & Hommel, 2009)	1.15	0.86 [0.63, 1.08]	10/13	14/17

The last columns provide sample sizes for the new effect size when aiming at a power of either 80% or 90% (paired *t*-test, *α* = .05). The two values in each column are for one-tailed and two-tailed tests, respectively

More precisely, Experiment 1 demonstrated an R-E compatibility effect (Kunde, 2001), usually interpreted as evidence for ideomotor effect anticipation. Experiments 3 and 6 were concerned with learning R-E associations over a longer time course (Exp. 3; Elsner & Hommel, 2001) and binding of R-E events from trial to trial (Exp. 6; Dutzi & Hommel, 2009). Experiments 2, 4, and 5 were concerned with event-file bindings of stimuli and responses. When a (spatial) feature was bound into an event file representing an action plan, concurrent perception was impaired for stimuli requiring the same (spatial) feature (Exp. 2; Müsseler & Hommel, 1997a, 1997b) and the same was true for concurrently executed motor actions (Exp. 5; Stoet & Hommel, 1999). Finally, Experiment 4 confirmed that stimuli and responses are bound into an event file once encountered, impairing subsequent performance when only parts of the implicated features were repeated in a subsequent event (Hommel, 1998).

These results are useful in at least two ways. First, we expect TEC to continue stimulating research in basic and applied areas. For researchers embracing TEC for one or the other reason, the present effect sizes can be used as informed starting points underlying their choice of sample size when conducting power analyses. Table 7 provides a summary of sample sizes required for 1 − *β* = .80 and 1 − *β* = .90 when using the simplest analysis tool, that is, a paired *t*-test. As a cautionary note, these numbers can change when the design is more complex and involves further independent variables (see, e.g., Brysbaert, 2019). Also, the calculated effect size was obtained with one particular experimental setting suited for data collection in the laboratory in this present study. Other settings may well yield larger effect sizes and thus allow a smaller sample size as well (see, e.g., the discussion of Exp. 1). When performing power analyses, it is thus useful to choose effect sizes from experimental settings that resemble the planned study as closely as possible. The values provided in Table 7 provide rules of thumb when an informed intuition about the size of the expected effect cannot draw on similar previous work.

The second contribution is that all six experiments replicated the main results reported in the original studies. Against the background of attempts to replicate certain psychological phenomena in the past (e.g., Open Science Collaboration, 2015), this outcome is certainly positive and encouraging news for the field. Considering recent calls for integrative frameworks going beyond phenomena-specific explanations (Eronen & Bringmann, 2021; Muthukrishna & Henrich, 2019; Oberauer & Lewandowsky, 2019) and cumulative theory building, TEC appears well-suited as being such a framework, backed up by solid evidence for core phenomena derived from it.

### Future directions

In our view, future research on TEC can extend the state of the art in two complementary directions that we label as vertical or horizontal.

With vertical extensions, we refer to research that aims at a deeper understanding of particular TEC-related phenomena with regard to potential underlying mechanisms. While TEC provides a coherent framework for understanding diverse empirical phenomena, many of these phenomena can also be captured by alternative accounts that do not necessarily accord with TEC’s perspective. For instance, findings on R-E learning (Exp. 3; Elsner & Hommel, 2001) have been suggested to reflect strategic choices (Vogel et al., 2018; Weller et al., 2017), possibly relying on propositional representations rather than direct action-effect associations (Sun et al., 2022). Having a precise grasp on the size of individual effects will allow for more stringent tests of potential alternative explanations, thus contributing to theoretical progress.

Horizontal extensions, by contrast, refer to the interrelation of different empirical effects. A major strength of TEC is its broad applicability across diverse phenomena in perception and action. With the present combined assessment of several empirical phenomena, we hope to contribute to research in this tradition. That is, the present re-assessment of selected phenomena should be especially valuable for building bridges between design-specific effects, possibly towards areas that are not commonly discussed in relation to TEC.

One example that would benefit from such cross-design approaches concerns the relation of action-induced blindness (Exp. 2; see Müsseler & Hommel, 1997a, 1997b) and sensory attenuation (Blakemore et al., 2000) as argued in the discussion of Experiment 2. Addressing how impaired perceptual processing either immediately before or immediately after performing an action builds on potentially similar mechanisms might allow for a more parsimonious explanation, while also highlighting shared mechanisms of TEC and common models that are invoked to explain sensory attenuation, especially forward and comparator models (Dogge et al., 2019; Horváth, 2015). Such research would also inform communities that have mainly relied on specific perceptual and response modalities, as is apparent in the decidedly visual focus of action-induced blindness, while at the same time drawing on modality-unspecific mechanisms to explain central observations. Finally, this strategy will also include the assessment of specific populations and corresponding inter-individual variation of certain phenomena in both clinical and non-clinical settings.

A second example concerns the relation of code occupation effects (Exp. 5, see Stoet & Hommel, 1999) and experiments that address the role of anticipated action effects for switching between different responses (Kunde et al., 2002; Mocke et al., 2020). On the surface, both strands of research seem to employ highly similar setups. Code occupation is commonly studied by having participants plan an action while performing an intermediate action that either shares features with the planned action or does not share any relevant features, with shared features yielding overlap costs. The alternative strand of research is identical in the sense that participants plan a certain movement, but have to perform a separate response while holding the initial action plan active. These studies also implement overlap conditions, but they do so by having responses produce either compatible or incompatible action effects in the actor’s environment, such as distinctive effect tones rather than in terms of body-related features as in typical setups that probe for code occupation. Strikingly, despite the highly similar setup, these latter studies have consistently found overlap benefits rather than overlap costs (see also Janczyk & Kunde, 2014). This change in direction of the effect appears puzzling and, therefore, requires future, cumulative evidence to arrive at a satisfactory account of both phenomena.

A third example of TEC’s broad applicability across different empirical phenomena is illustrated by recent trends to study well-established cognitive effects in social settings, that is, when two or more individuals engage together in a cognitive task. Interestingly, many experiments now come with a social variant (e.g., the *joint Simon task*, Sebanz et al., 2003; *joint flanker task*, Atmaca et al., 2011; *social inhibition of return*, Janczyk et al., 2016; *observational R-E associations*, Paulus et al., 2011; Pfister et al., 2014a; *observational S-R binding*, Giesen et al., 2014, to name just a few). On the one hand, these developments illustrate that these well-established effects are not immune to social influences, an insight that is only recently recognized by cognitive psychologists. On the other hand, the findings from these new approaches set the stage to introduce the terminology of TEC to account for social effects. This allows for re-assessing prominent effects known from social psychology and explaining them at least partly from TEC’s perspective (for recent examples, see Giesen et al., 2014, 2017, 2018; Hommel & Colzato, 2015; Hommel & Stevenson, 2021).

In sum, the present re-assessment of selected phenomena should be especially valuable for building bridges between design-specific effects, possibly towards areas that are not commonly discussed in relation to TEC. Not surprisingly, in the two decades after their introduction, the basic ideas of TEC have been further developed and extended beyond those paradigms closely associated with TEC. For example, the recent *Binding and Retrieval in Action Control* (BRAC) framework explicitly separates integration (i.e., binding) and retrieval processes theoretically and proposes that these two processes affect action control also in many standard experiments, typically not closely associated with event coding (e.g., negative priming, task switching, Pavlovian conditioning, visual search; Frings et al., 2020). Such broad applicability of the processes involved in the current experiments, is another reason to expect continued research interest in TEC-related phenomena in the future.

## Conclusions

The present study reported six experiments on phenomena that have been judged important for understanding human perception and action from the perspective of TEC (Hommel et al., 2001). Importantly, all experiments were run with a large sample size to provide realistic estimates of effect sizes with narrow confidence intervals. We believe, this approach advances theory building and provides a solid background for future studies on TEC and its related phenomena.

## Figures and Tables

**Fig. 9:**
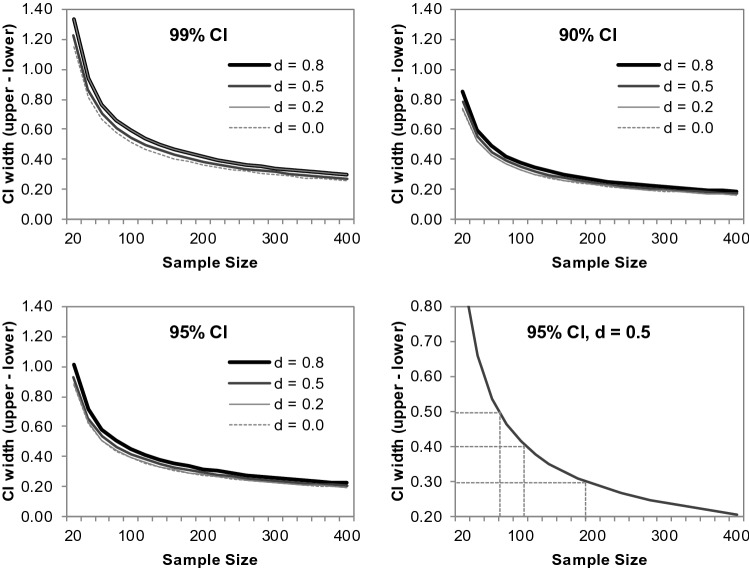
Widths of different confidence intervals for standardized effect sizes as a function of the underlying population effect size and the sample size of an empirical study. All estimates were computed with help of the MBESS package for R (Kelley, 2007)

## Data Availability

Data and analyses scripts can be found at https://osf.io/hgy5q/. Experiments and analyses were pre-registered at: https://aspredicted.org/ad6xq.pdf.

## Appendix A

The following form was sent in September 2018 to 114 experts on (basic) research in the domain of perception and action, and answers were collected for one month. Experts were invited via email and provided their response via a Google form. To arrive at a maximally representative opinion, we further asked each expert to suggest additional researchers that we might have missed in our initial list.

The questionnaire contained a brief introductory text and two questions as follows. Answers were provided by tick-marking each paradigm (section “Experimental paradigms”) and in terms of free text (section “Comments”).

### TEC: empirical approaches

Please rate the following experimental paradigms based on their relevance for the study of action control from the perspective of the Theory of Event Coding (TEC; Hommel et al., 2001).

To do so, tick all following paradigms which you perceive as closely tied to TEC, either by lending considerable support to the theoretical notions of TEC, or by being immediately stimulated by the theory.

### Experimental paradigms

- Action-induced blindness (e.g., Müsseler & Hommel, 1997a, 1997b, JEP:HPP).
- Code occupation (e.g., Stoet & Hommel, 1998, JEP:HPP).^9^
- Dimension weighting (e.g., Fagioli et al., 2007, PRPF).
- Distractor-response binding (e.g., Frings et al., 2007, QJEP).
- Feature weighting (e.g., Memelink & Hommel, 2013, PRPF).
- Response-effect compatibility (e.g., Kunde, 2001, JEP:HPP).
- Response-effect learning (e.g., Elsner & Hommel, 2001, JEP:HPP).
- Sensory attenuation (e.g., Blakemore et al., 2000, Neuroreport).
- Short-term response-effect binding (e.g., Dutzi & Hommel, 2009, PRPF).
- Stimulus–response binding (e.g., Hommel, 1998, Visual Cognition).

### Comments

If you feel that the above list misses out on a critical paradigm, or if you would like to share other comments, please tell us in the field below.

## Appendix B

We planned our sample size according to the predicted width of the resulting confidence interval around each effect size estimate. Figure 9 shows the resulting width of 99%, 95%, and 90% confidence intervals for standardized means for different population effect sizes as a function of the sample size. We eventually settled on a 95% confidence interval and an estimated effect size of Cohens *d*_*z*_ = 0.5. Note that the impact of the population effect size is only modest so that corresponding estimates also generalize to other effect sizes. For the chosen setting, confidence intervals with a width of 0.5 would require sample sizes of around *n* = 80; a width of 0.4 results for studies of *n* = 100, whereas a width of 0.3 would require roughly *n* = 200. We therefore opted for a precision of 0.4 as the added precision of 0.3 would incur a disproportionally high cost.

## Appendix C

All effect sizes were computed based on the test statistics and sample sizes reported in the original articles. We then used this information to compute 95% confidence intervals for standardized means using the ci.sm() function of the MBESS package (Kelley, 2007). The corresponding effect size calculations were:

- Response-effect compatibility (Kunde, 2001): ${d}_{z}=\frac{\sqrt{F}}{\sqrt{n}}=\frac{\sqrt{9.28}}{\sqrt{10}}=0.96$
- Action-induced blindness (Müsseler & Hommel, 1997a): ${d}_{z}=\frac{t}{\sqrt{n}}=\frac{2.30}{\sqrt{14}}=0.61$
- Response-effect learning (Elsner & Hommel, 2001): ${d}_{z}=\frac{t}{\sqrt{n}}=\frac{3.25}{\sqrt{12}}=0.94$
- Stimulus–response binding (Hommel, 1998): ${d}_{z}=\frac{\sqrt{F}}{\sqrt{n}}=\frac{\sqrt{58.18}}{\sqrt{8}}=2.70$
- Code occupation (Stoet & Hommel, 1999): ${d}_{z}=\frac{\sqrt{F}}{\sqrt{n}}=\frac{\sqrt{18.42}}{\sqrt{18}}=1.01$
- Short-term response-effect binding (Dutzi & Hommel, 2009): ${d}_{z}=\frac{t}{\sqrt{n}}=\frac{4.86}{\sqrt{18}}=1.15$

## Footnotes

1 Of course, other theoretical frameworks exist that also deal with perception and action, such as, for example, the Perception–Action Model of visual processing (Milner & Goodale, 2006). The present focus on TEC in the theoretical parts of the article derives from the background of this work as seeking to re-assess critical observations that either stimulated the formulation of TEC or that were directly influenced by this theoretical framework.

2 According to Descartes (1664), muscle movements result from sensorial input stimulating the pineal gland, which served as a central hub for cognition, motivation, and volition in his view. Upon deciding on a bodily movement, it would pour liquids into the muscles to move them.

3 The terms “proximal” and “distal” are sometimes also used to refer to intrinsic events concerning an agent’s body versus extrinsic events in the external environment (e.g., Jordan, 2013; Ladwig et al., 2012). In fact, early formulations of TEC also switched between the body-environment meaning of the terms (e.g., "distal, “extrinsic”, action-contingent events"; Hommel et al., 2001, p. 868) and their current meaning, whereas the meaning described above was made explicit in later publications on TEC (Hommel, 2009).

4 Data were collected between September 2019 and November 2020. Data collection was suspended for some time, because of the lockdown during the COVID-19 pandemic that applied to behavioral laboratories at many universities.

5 We report the quintile analysis mainly to remain consistent with the analysis in the original publication (Kunde, 2001). Note that increasing effects with longer RTs often reflect the statistical relation of mean RT and the corresponding standard deviation (Proctor et al., 2011; Zhang & Kornblum, 1997). The observed pattern is therefore non-diagnostic and it is also predicted by many RT models (see Wagenmakers & Brown, 2007). The most well-known exception from this general pattern is the visual Simon effect which becomes smaller with longer RTs (Burle et al., 2005; Pratte et al., 2010).

6 Note that Janczyk and Lerche (2019) reported a slightly different estimate of this effect size. Their value was based on the actual raw data of Kunde (2001), whereas we preferred to estimate the effect size based on the test statistic reported in the original article to use a consistent approach across all experiments of the present study.

7 In the original experiment, eight familiarization trials were used. During the following acquisition trials, participants could take self-paced breaks by pressing the escape key, and the “response ratio” was presented to the participants following 50 valid acquisition trials.

8 Whether or not a pattern of results “replicates” a previous observation requires a subjective appraisal of the data at hand (Open Science Collaboration, 2015). We do not see the consistent reduction in effect sizes as critical in that regard, because most theoretical arguments in the relevant literature are not based on the magnitude, but are rather based on the direction of observed effects. A second point worth commenting is the two-tailed confidence interval for the action-induced blindness effect tested in Experiment 2. This interval included 0 whereas a one-tailed test still yielded a significant effect. Moreover, the present effect size estimate was included in the confidence interval for standardized means of the original publication. Against this background, we believe it pertinent to assume that this effect likely exists, albeit being very small.
